# Novel monoclonal antibody L2A5 specifically targeting sialyl-Tn and short glycans terminated by alpha-2–6 sialic acids

**DOI:** 10.1038/s41598-018-30421-w

**Published:** 2018-08-15

**Authors:** Liliana R. Loureiro, Diana P. Sousa, Dylan Ferreira, Wengang Chai, Luís Lima, Carina Pereira, Carla B. Lopes, Viviana G. Correia, Lisete M. Silva, Chunxia Li, Lúcio Lara Santos, José Alexandre Ferreira, Ana Barbas, Angelina S. Palma, Carlos Novo, Paula A. Videira

**Affiliations:** 1 UCIBIO-REQUIMTE, Department of Life Sciences, Faculty of Science and Technology, NOVA University of Lisbon, Lisbon, 2829 Portugal; 2 iBET, Instituto de Biologia Experimental e Tecnológica, Oeiras, 2780 Portugal; 3 Experimental Pathology and Therapeutics Group, IPO-Porto Research Center, Portuguese Institute of Oncology of Porto, Porto, 4200 Portugal; 4 Glycosciences Laboratory - Department of Medicine, Imperial College London, London, W12 0NN United Kingdom; 5 Glycobiology in Cancer, Institute of Molecular Pathology and Immunology of the University of Porto (IPATIMUP), Porto, 4200 Portugal; 6 Institute for Research and Innovation in Health (I3S), University of Porto, 4200 Porto, Portugal; 7 CINTESIS - Center for Health Technology and Services Research, University of Porto, Porto, 4200 Portugal; 8 Molecular Oncology and Viral Pathology Group, IPO-Porto Research Center, Portuguese Oncology Institute of Porto, Porto, 4200 Portugal; 9 Joaquim Chaves Saúde, Anatomical Pathology Laboratory, Lisboa, 1170 Portugal; 10 UCIBIO-REQUIMTE, Department of Chemistry, Faculty of Science and Technology, NOVA University of Lisbon, Lisbon, 2829 Portugal; 11 Key Laboratory of Marine Drugs of Ministry of Education, and Shandong Provincial Key Laboratory of Glycoscience and Glycoengineering, School of Medicine and Pharmacy, Ocean University of China, Qingdao, 266003 China; 12 Institute of Biomedical Sciences Abel Salazar, University of Porto, Porto, 4050 Portugal; 13 Department of Surgical Oncology, Portuguese Institute of Oncology, Porto, 4200 Portugal; 14 International Iberian Nanotechnology Laboratory (INL), Braga, 4715 Portugal; 15 Bayer Portugal, Carnaxide, 2790 Portugal; 16 UEIPM, Institute of Hygiene and Tropical Medicine, NOVA University of Lisbon, Lisbon, 1349 Portugal; 17 CDG & Allies – Professionals and Patient Associations International Network (CDG & Allies – PPAIN), Caparica, 2829 Portugal

## Abstract

Incomplete *O*-glycosylation is a feature associated with malignancy resulting in the expression of truncated glycans such as the sialyl-Tn (STn) antigen. Despite all the progress in the development of potential anti-cancer antibodies, their application is frequently hindered by low specificities and cross-reactivity. In this study, a novel anti-STn monoclonal antibody named L2A5 was developed by hybridoma technology. Flow cytometry analysis showed that L2A5 specifically binds to sialylated structures on the cell surface of STn-expressing breast and bladder cancer cell lines. Moreover, immunoblotting assays demonstrated reactivity to tumour-associated *O*-glycosylated proteins, such as MUC1. Tumour recognition was further observed using immunohistochemistry assays, which demonstrated a high sensitivity and specificity of L2A5 mAb towards cancer tissue, using bladder and colorectal cancer tissues. L2A5 staining was exclusively tumoural, with a remarkable reactivity in invasive and metastasis sites, not detectable by other anti-STn mAbs. Additionally, it stained 20% of cases of triple-negative breast cancers, suggesting application in diseases with unmet clinical needs. Finally, the fine specificity was assessed using glycan microarrays, demonstrating a highly specific binding of L2A5 to core STn antigens and additional ability to bind 2–6-linked sialyl core-1 probes. In conclusion, this study describes a novel anti-STn antibody with a unique binding specificity that can be applied for cancer diagnostic and future development of new antibody-based therapeutic applications.

## Introduction

Cancer is one of the most serious public health problems worldwide with a major impact on society. The American Cancer Society report for 2017 estimates that 1 688 780 new cancer cases and 600 920 cancer deaths are predicted to occur in the United States, identifying this condition as an enormous burden mainly for developed countries^1^. Despite significant progress in traditional methods of cancer therapy, such as chemotherapy, radiation and surgery, cancer remains an extremely difficult disease to cure or prevent. As such, the quest for novel therapies against this disease became crucial in the last decades.

**Figure 1:**
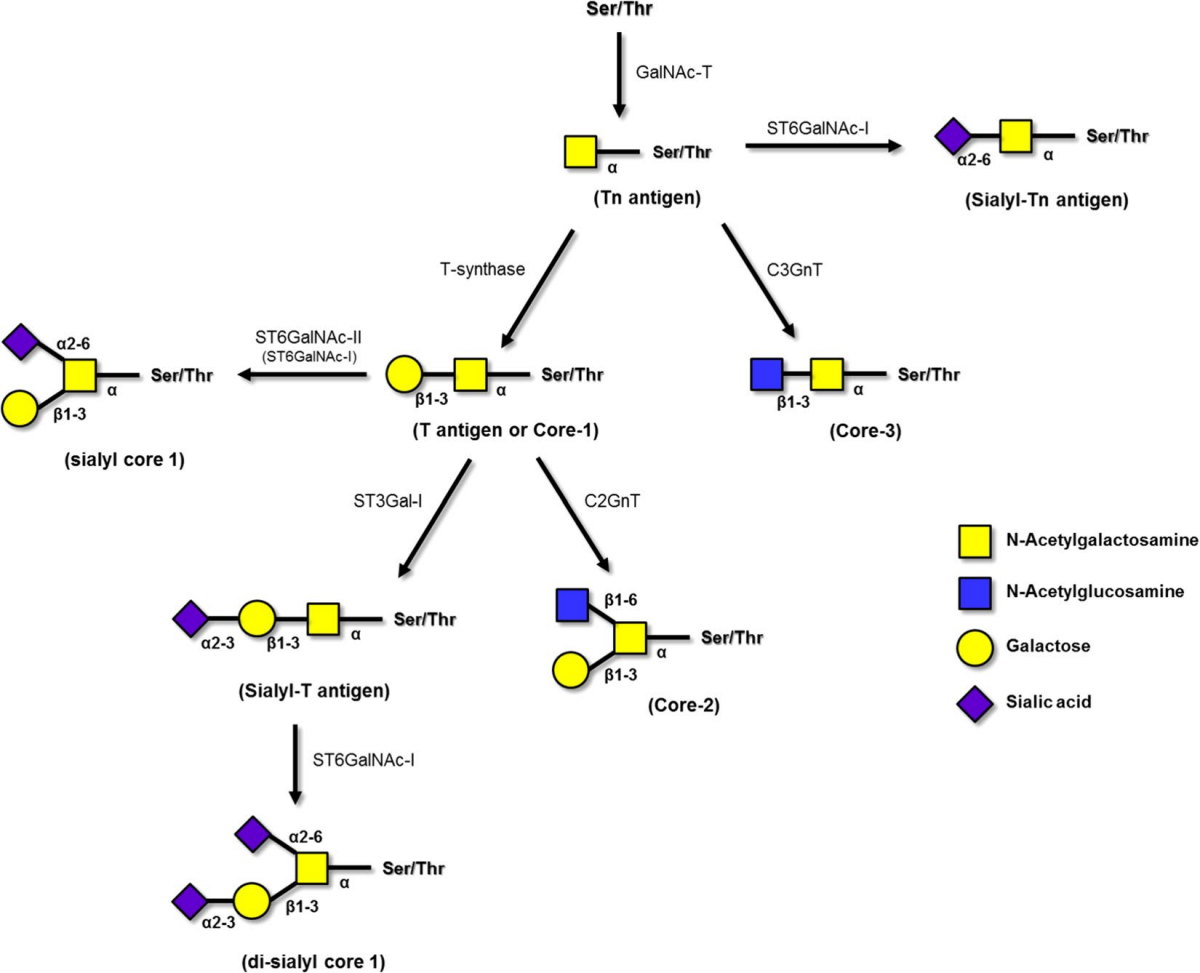
Biosynthesis of *O*-glycan chains. *O*-glycosylation is initiated by the transfer of a GalNAc residue to a serine or threonine residue present on a polypeptide by peptidyl-*N*-acetylgalactosaminyltransferases (GalNAc-T) creating a Tn antigen. This antigen can be further modified by ST6GalNAc-I leading to the development of Neu5Acα2–6GalNAcSer/Thr known as STn antigen, by T-synthase to T antigen or core-1, or by C3GnT forming core-3 structures. Furthermore, the T antigen can be additionally converted by C2GnT to core-2 structures or sialylated by ST3Gal-I creating sialyl-T antigens. The monosaccharides are depicted according to Symbol Nomenclature for Graphical Representation of Glycans^65^. Adapted from references^29,30^.

Glycosylation is a biological process that allows the sequential covalent addition of specific glycan structures to the backbone of lipids and proteins. This process is commonly observed in cancer, resulting in the expression of aberrant tumour-associated carbohydrate antigens (TACA). TACA plays a key role in tumour progression regulating processes such as cellular adhesion, angiogenesis, signalling and recognition mechanisms and importantly, tumour invasion and metastasis^2–5^. Incomplete *O*-glycosylation is one of the features associated with malignancy leading to the expression of TACA such as truncated *O*-glycans, namelly the Thomsen-nouveau (Tn; GalNAcα1-*O*-Ser/Thr) antigen and its sialylated version the sialyl-Tn antigen (STn; Neu5Acα2–6GalNAc linked to a serine or threonine residue (Ser/Thr)). Particularly, STn results from an early sialylation of the Tn antigen. This is due to overexpression of the enzyme STn synthase (ST6GalNAc-I) (Fig. 1)^4–7^ and/or disruption of further elongation processes^4,8–10^ due to mutations in the molecular chaperone Cosmc that provides proper folding and activity of the T-synthase^11,12^. The Tn antigen can be elongated with galactose to form the T antigen (also known as Core 1 or Thomsen-Friedenreich antigen). *O*-glycans in healthy cells are generally extended to complex branched Core 2, 3 or 4 structures, while in cancer are truncated with the linkage of sialic acid forming in addition to the STn antigen, also the α2–6 sialyl-T, the sialyl-T and the disialyl-T antigens (Fig. 1). STn is negligible expressed in healthy tissues but overexpressed in more than 80% of the human carcinomas, such as bladder^13^, ovarian^14^, colon^15,16^, breast^17^ and prostate^18–20^ cancers. STn modulates a malignant phenotype associated with a poor prognosis in cancer patients^21–24^, chemotherapy resistance^25^ and induction of a tolerogenic phenotype on immune cells^26^. Therefore, this antigen is considered a relevant target for the development of anti-cancer immunotherapeutic strategies and vaccines^27,28^.

Targeted therapy based on antibodies is a rapidly progressing field and has the promising potential to enhance immune responses against STn-expressing tumour cells^26,29,30^. The use of monoclonal antibodies (mAbs) for therapeutic purposes has broadly increased in the last decades, with around 47 mAb products being approved in the United States or Europe^31,32^. As most of TACAs, STn does not elicit strong humoral responses due to inefficient T cell activation^26^. Several anti-STn mAbs have been developed using a variety of STn^+^ immunogens, thus presenting different specificities and affinities to STn positive tumour cells^30^. MAbs such as B72.3, TKH2 and 3F1 are some examples of anti-STn antibodies initially developed and mainly used for diagnostic purposes^33,34^. In addition, an IgM mAb 3P9 was reported to directly inhibit tumour growth^35^. More recently, antibodies specifically targeting STn glycan have been reported and their conjugation with drugs led to the development of antibody-drug conjugates that demonstrated *in vitro* efficacy in STn-expressing cell lines and tumour growth inhibition in tumour xenograft cancer models^36^.

Despite all the progress in the development of anti-STn mAbs with a potential therapeutic application, target specificity has come into question as they differ in their ability to bind additional glycan targets and in their preferences regarding antigen recognition based on carrier proteins. In this study, we report the development of a novel anti-STn IgM mAb L2A5 using hybridoma technology. Thorough characterization assays demonstrate the specificity of this antibody to STn, binding to short α-2–6 sialyl mucin *O*-glycan core-1 glycan sequences, and its potential to discriminate tumour tissue.

## Results

### Development and screening of anti-STn mAbs

**Figure 2:**
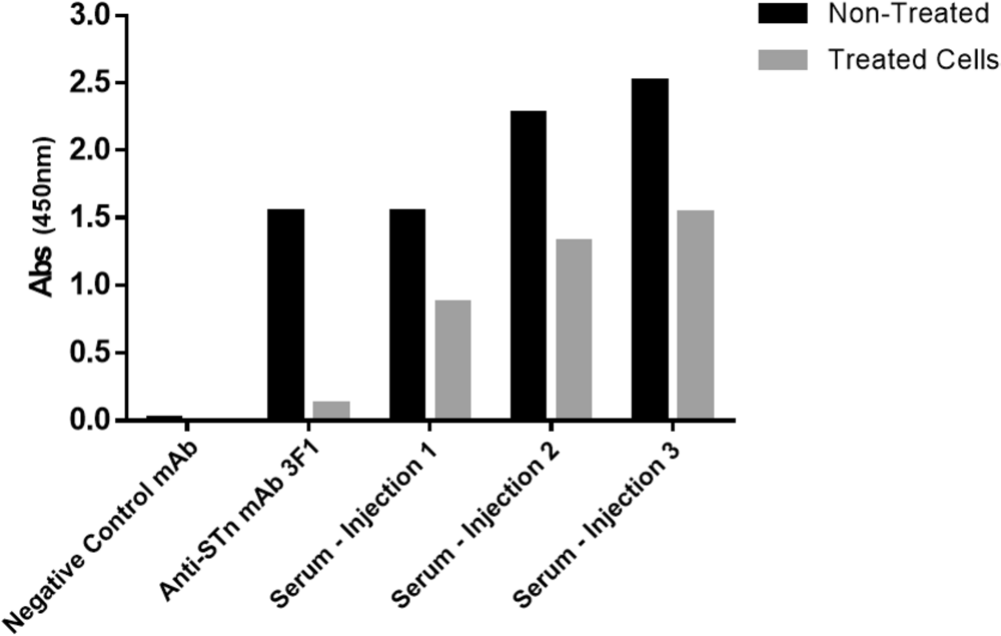
Assessment of the presence of antibodies in mouse serum with ability to bind to sialylated structures in BSM determined by indirect ELISA. Mice were immunized with three injections of 10 µg of OSM, with 21 days interval. Serum was collected and analysed after each injection for the presence of antibodies targeting sialylated structures. An antibody non- specific for sialylated structures was used as negative control and the anti-STn mAb 3F1 was used as positive control. Cells without sialidase treatment (non-treated cells) and cells pre-treated with sialidase (treated cells) were used.

In this study, we aimed to develop and characterize a novel anti-STn antibody. For that, mice were immunized with ovine submaxillary mucin (OSM), a protein in which 98% of the glycan content is STn^37^. Blood samples were collected, and serum was regularly tested to monitor animal immunization and to identify the optimal timing for splenic collection. After each injection, mouse serum was collected and analysed for the presence and titer of antibodies binding to sialylated structures on bovine submaxillary mucin (BSM) surface using indirect ELISA (Fig. 2). Splenocytes from immunized mice presenting the highest antibody titer were then fused with Sp2/0 myeloma cells^38^ and hundreds of hybridomas were generated. Stable hybridomas were established by successive limiting dilution cloning, and their supernatants were screened for the presence of antibodies with reactivity towards STn antigens and STn^+^ cells using ELISA and flow cytometry, respectively. Selected hybridomas were expanded for antibody production and characterization. From the several anti-STn mAbs obtained, mAb L2A5 was selected as the lead candidate and further characterized using different techniques.

### L2A5 mAb isotyping and titer determination

**Figure 3:**
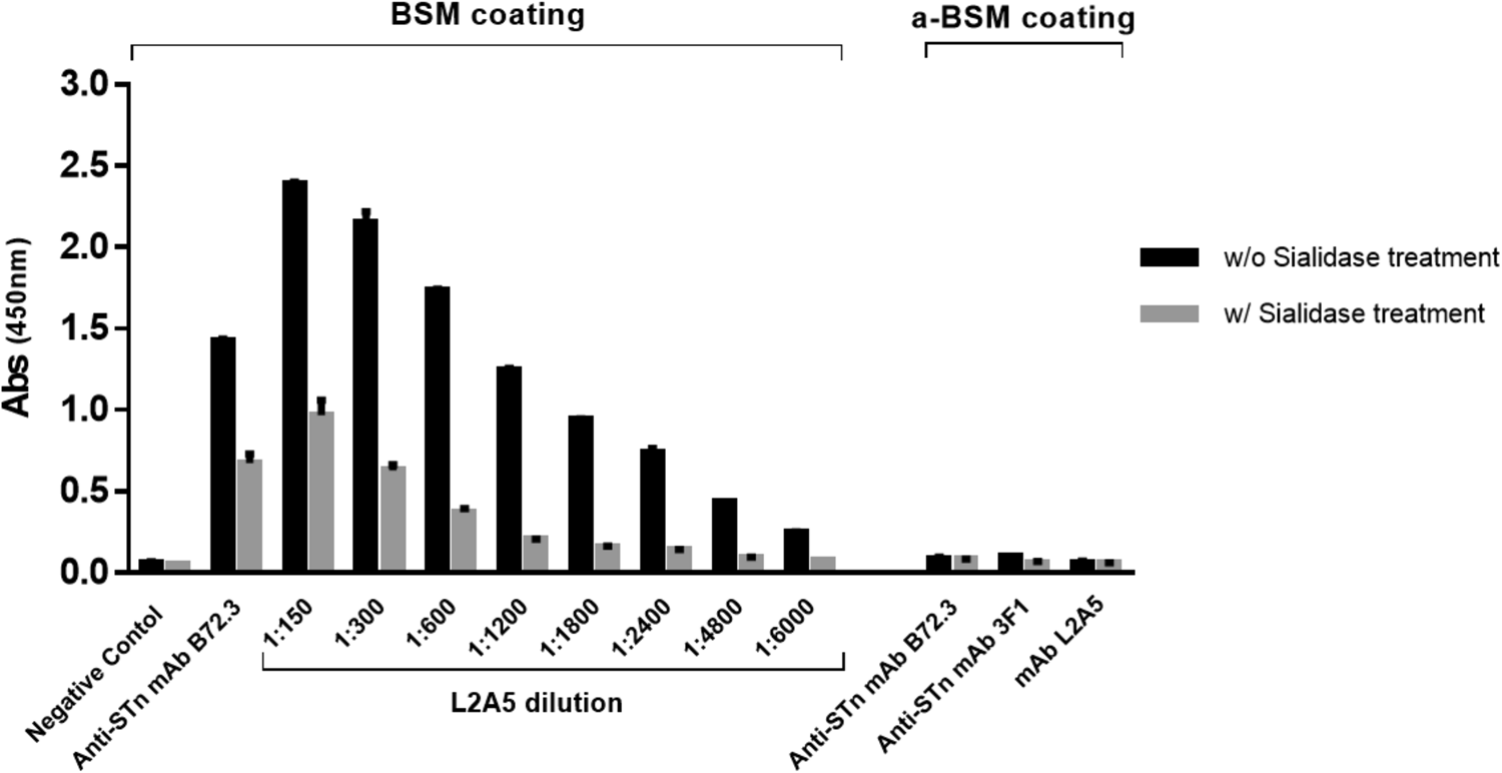
Measurement of L2A5 mAb titer and binding assessment to BSM and asialo-BSM using indirect ELISA. Antibody titration was performed using various dilutions of L2A5 mAb in PBS and BSM as coating antigen. BSM was used either without sialidase treatment (black bars) or pre-treated with sialidase (grey bars). In addition, binding assessment of anti-STn mAbs was performed using asialo-BSM (a-BSM) as coating antigen. PBS was used as negative control and anti-STn mAbs B72.3 and 3F1 as positive controls. Control antibodies and L2A5 mAb used in the binding assessment to a-BSM were diluted 1:1000. Antibody binding was detected with goat anti-mouse Ig-HRP.

**Figure 4:**
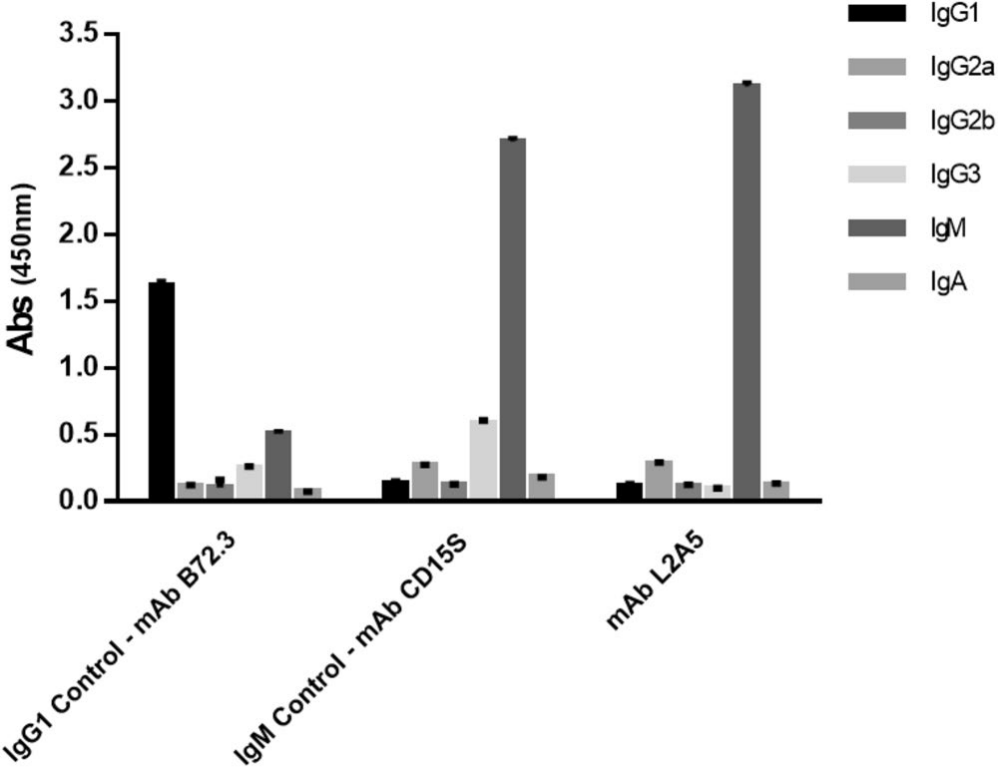
L2A5 isotype determination using capture ELISA. 1:1000 dilution of L2A5 mAb was used in a capture ELISA with IgG1, IgG2a, IgGb, IgG3, IgM and IgA. Results are expressed as mean of absorbance (Abs) at 450 nm + standard deviation. Antibodies with known isotype B72.3 (IgG1) and CD15S (IgM) were used as controls (CTR).

The reactivity and antibody titer of the L2A5 mAb was determined by indirect ELISA as shown in Fig. 3. BSM was used as target antigen since it predominantly contains STn moieties as well as to decrease the possibility of obtaining mAbs only with reactivity against the immunogen OSM^39^. The reactivity response curve was drawn by plotting absorbance against serial dilutions of L2A5 mAb. As displayed in Fig. 3, there is a logarithmic dependence between absorbance and mAb concentrations, reaching the endpoint titer of 1:6000. Furthermore, BSM treatment with sialidase demonstrated a reduction in the reactivity of this antibody, revealing the specific and dose-dependent binding to sialylated structures, most likely the STn antigen (Fig. 3, grey bars). Nevertheless, since the sialidase treatment may not completely remove the sialic acid residues present on the surface of the protein, we performed an indirect ELISA using BSM deprived of STn antigens (asialo-BSM) as coating antigen. As expected, no reactivity by the L2A5 mAb was observed against this molecule (Fig. 3). Isotype determination using IsoStrip™ Mouse Monoclonal Antibody Isotyping Kit (data not shown) and isotype capture ELISA (Fig. 4) determined that L2A5 is an IgM mAb with κ light chains. Furthermore, L2A5 mAb was purified and characterized regarding its affinity, for which an equilibrium dissociation constant (K_D_) value of 2.25 nM was determined using bio-layer interferometry, a label-free technology for measuring biomolecular interactions (see Supplementary Fig. S1).

### Recognition of STn by L2A5 mAb in different cancer cell lines using flow cytometry

**Figure 5:**
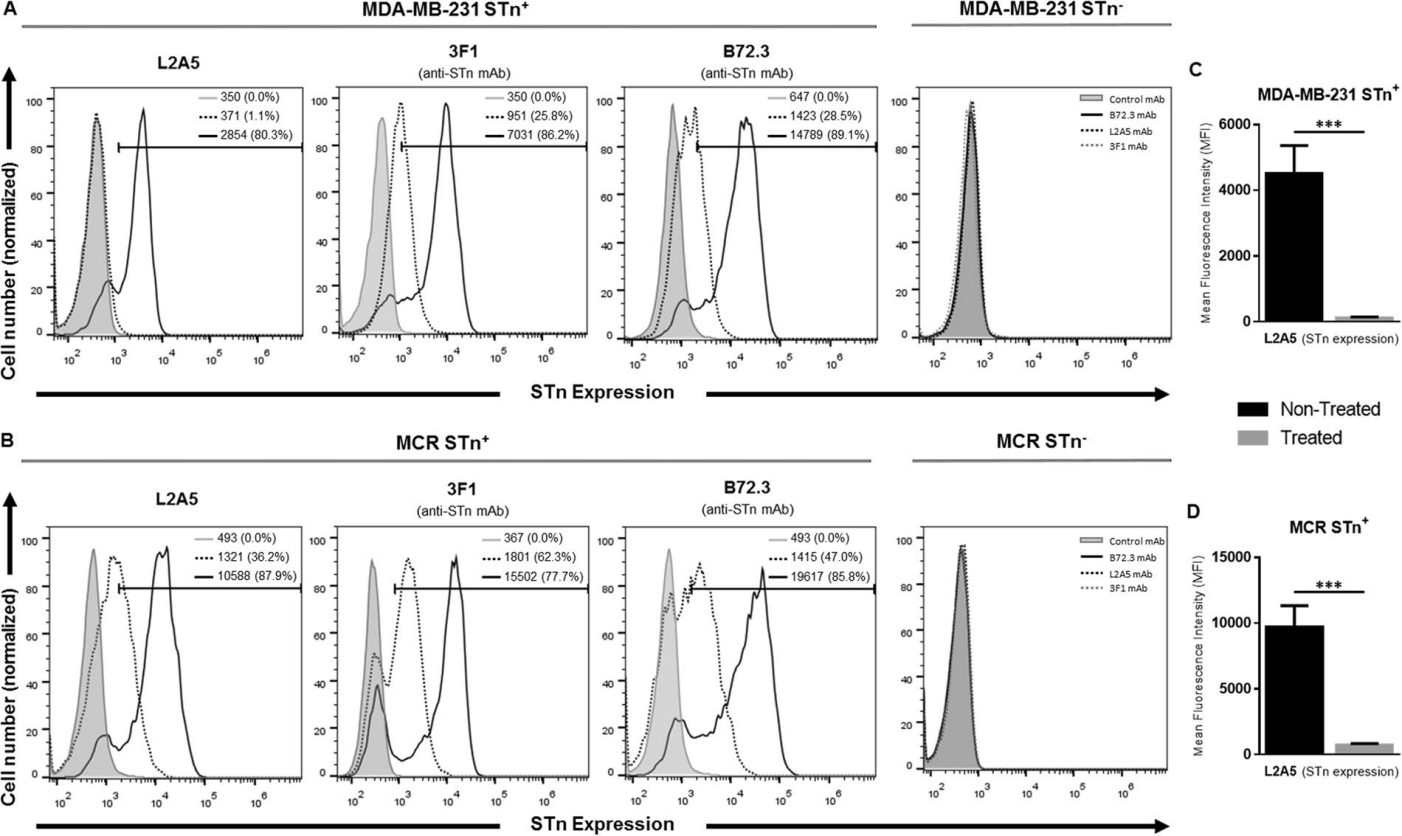
L2A5 and anti-STn mAbs binding assessment to STn^+^ and STn^−^ cancer cells using flow cytometry. Representative histograms of cell count (normalized) versus the mean fluorescent intensity (MFI) obtained for L2A5, B72.3 or 3F1 mAbs (anti-STn control antibodies) and control antibody (secondary anti-mouse Ig-FITC mAb – grey profile). For the assessment of STn binding, the breast cancer cell line MDA-MB-231 (**A**) or the bladder cancer cell line MCR (**B**) STn positive or negative (STn^+^ or STn^−^) were used (see also Materials and Methods). To assess binding specificity to STn antigen, STn-expressing cell lines were desialylated by sialidase treatment. The control antibody is represented as filled grey area, whereas solid and dashed lines represent the histograms for non-treated and sialidase treated cells, respectively. Numbers represent percentage of positive cells and MFI of total cells for each condition. (**C**,**D**) Column plots showing the mean ± standard deviation from 3 independent experiments of the flow cytometric analysis using L2A5 mAb to stain MDA-MB-231 STn^+^ (**C**) and MCR STn^+^ (**D**) cells, which were (treated) or not pre-treated (non-treated) with sialidase. MFI of stained samples was normalized to the MFI value of the negative control. Statistical analysis was performed using Student’s t-test (***p < 0.001).

Binding of the mAb L2A5 to the cell surface of STn^+^ breast cancer cells MDA-MB-231 and STn^+^ bladder cancer cells MCR was evaluated by flow cytometry. To assess the sialic acid-dependent binding, STn^+^ cancer cells were treated with sialidase^40^ and binding to non-treated and sialidase treated cells was determined. The results show that the L2A5 mAb binds to a high percentage of cells, 80 to 88%, on both non-treated STn^+^ MDA-MB-231 and MCR cells (Fig. 5A,B). Contrarily, a considerable decrease of this percentage was observed for the binding performed using the same cell lines pre-treated with sialidase. L2A5 demonstrated negligible binding to sialidase treated MDA-MB-231 STn^+^ cell line and bound to approximately 35% of sialidase treated MCR STn^+^ cells (Fig. 5A,B). Accordingly, significant decrease of mean fluorescence intensity (MFI) values were observed when STn^+^ cells were treated with sialidase, corroborating the sialic acid-dependent binding of the mAb L2A5 (Fig. 5C,D). In contrast and as expected, no binding was observed on corresponding STn-negative cancer cell lines (Fig. 5A,B). Anti-STn mAbs 3F1 and B72.3 were used as control antibodies for the assessment of STn expression and sialidase treatment. As shown in Fig. 5, 3F1 and B72.3 mAbs present similar binding patterns to both cancer cell lines as well as a similar decrease in the binding to sialidase treated cells. Conversely, while binding of L2A5 to MDA-MB-231 STn^+^ treated with sialidase is negligible, binding of B72.3 and 3F1 mAbs to cells treated with sialidase is observed with values around 26% (Fig. 5A). L2A5 binding curves for MCR STn^+^ cells resemble the binding patterns of the anti-STn control mAbs, with slightly lower percentage and MFI values (Fig. 5B). Taken together, these results suggest that the mAb L2A5 mainly binds to sialylated antigens, most likely to the STn antigen, present on the surface of cancer cells. Furthermore, these data highlight the different binding patterns obtained in comparison with other commonly used anti-STn mAbs, such as 3F1 and B72.3.

### Binding of L2A5 mAb to STn^+^ proteins and membrane extracts using western blot

**Figure 6:**
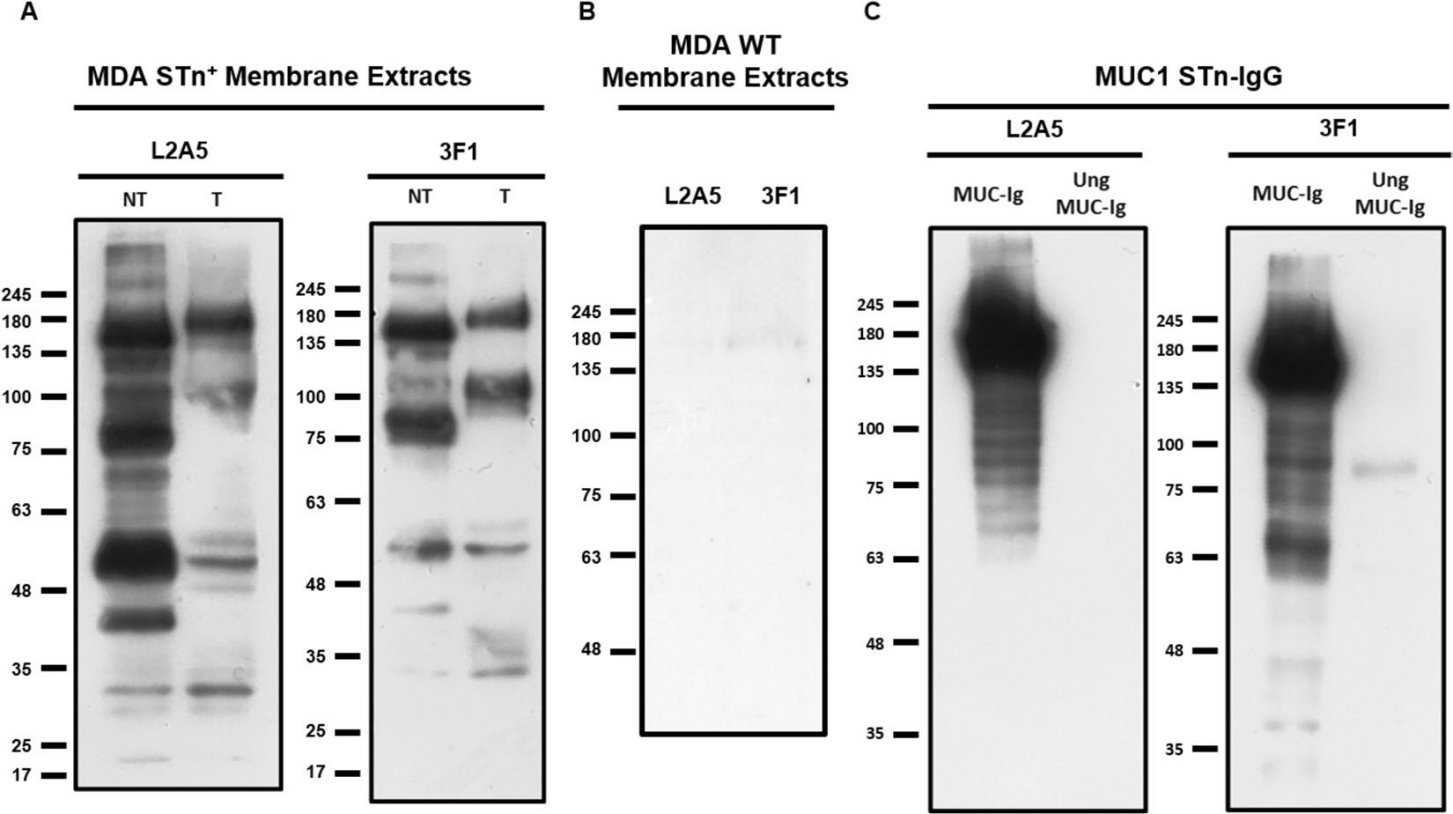
Western blot analysis of anti-STn mAbs binding to STn-expressing cell lines and MUC1 STn – IgG. MDA-MB-231 STn^+^ (**A**) and STn^−^ (**B**) membrane extracts, as well as the chimeric protein MUC1 STn-IgG (**C**) were stained with L2A5 and 3F1 mAbs. Blotting was performed using non-treated membrane extracts (NT) and chimeric protein (MUC-Ig) as well as membrane extracts samples that were desialylated using sialidase (T) or unglycosylated MUC1 STn-IgG protein (Ung MUC-Ig). Representative western blots (cropped) are shown. Full length western blots are provided in Supplementary Fig. S2.

To confirm the binding specificity of L2A5 mAb to STn^+^ proteins, we performed a western blot analysis of cancer cell- derived proteins. For that, MDA-MB-231 STn^+^ membrane extracts non-treated and treated with sialidase, as well as the chimeric protein MUC1 STn-IgG and its unglycosylated version were used. As shown in Fig. 6 (see also Supplementary Fig. S2), the L2A5 antibody binds to residues present in cell membrane extracts and in the MUC1 STn-IgG, and overall, this binding was significantly reduced after sialidase treatment. Concerning the membrane extracts samples, the molecular weight of the main bands detected using L2A5 were above 245 kDa and approximately 160, 85, 50 and 40 kDa. Upon desialylation, a decrease or abolishment of staining was observed (Fig. 6A). In the case of MUC1 STn-IgG, a robust binding was observed to a protein with approximately 180 kDa, which is in accordance with the molecular weight of the MUC1 molecule (Fig. 6C). As expected, no binding was observed using unglycosylated MUC1 STn-IgG due to the absence of STn antigens. Furthermore, no bands were detected on the control membrane extracts of MDA-MB-231 STn^−^ cancer cells (Fig. 6B). The anti-STn mAb 3F1 presented a similar binding pattern for both cell membrane extracts and MUC1 STn-IgG. Taken together, the results indicate that L2A5 mAb recognizes the STn-antigen in membrane extracts of STn-expressing cancer cells, as well as on STn carrier proteins such as MUC1.

### Cancer tissue specificity using immunohistochemistry

**Figure 7:**
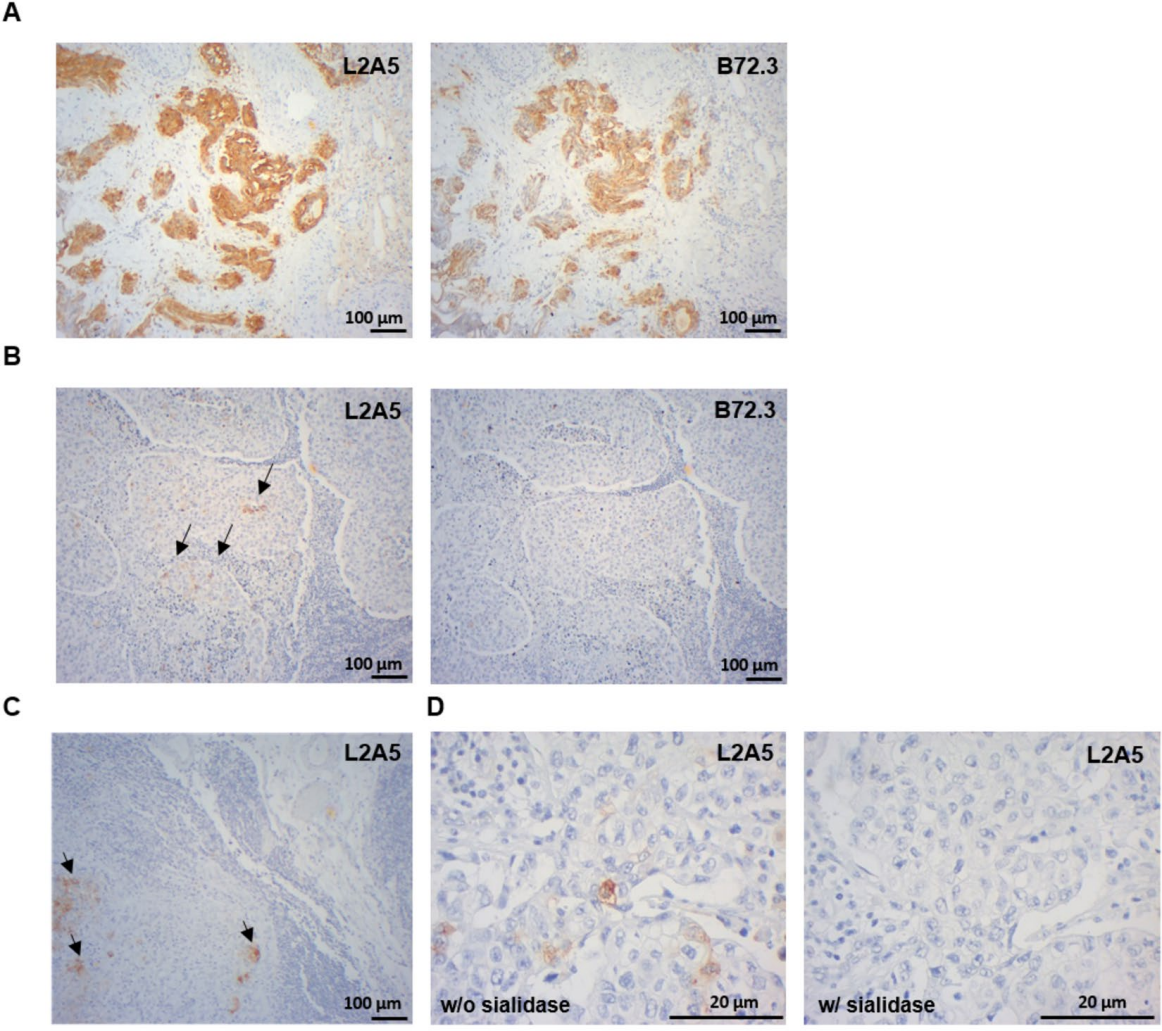
Immunohistochemical evidence of L2A5 mAb higher sensitive staining in bladder cancer samples. (**A**) Immunohistochemistry staining for L2A5 mAb in bladder cancer tissues. Sequential paraffin embedded tissue sections were stained with L2A5 and B72.3 mAb demonstrating the different staining patterns obtained. Higher extension and stronger signal intensity were observed using L2A5 mAb. (**B**) Immunohistochemistry of low-grade bladder cancer tissues. Sequential paraffin embedded tissue sections were stained with L2A5 and B72.3 mAb. Representative images show the increased sensitivity of L2A5 mAb (arrows) compared to lack of staining using B72.3. (**C**) Immunohistochemistry of metastatic bladder cancer tissue using L2A5 mAb. Staining of paraffin embedded tissue shows specific tumour cell staining (arrows) and absence of staining in lymphocytic cells, vessels and connective tissue. (**D**) Sialic acid-dependence of L2A5 staining. Immunohistochemistry staining using L2A5 was performed in non-treated and sialidase treated paraffin embedded bladder cancer tissue. Representative images show complete absence of staining after sialidase treatment.

The binding specificity of L2A5 mAb to cancer tissues was further assessed using immunohistochemistry on a series of 15 bladder tumours (eight muscle-invasive bladder cancer (MIBC) tumours and seven ganglionar metastasis), 14 colorectal tumours (adenocarcinomas and adenomas) and 5 normal colorectal mucosa samples. The anti-STn mAb B72.3 was used for comparison. All eight primary bladder tumours were positive for all the analysed mAbs. The L2A5 mAb exhibited an increased sensitivity for antigen detection in bladder cancer cases, including higher extension and stronger signal intensity (approximately 75% of the cases) when compared to B72.3 (Fig. 7A, Table 1). In addition, L2A5 staining was exclusively tumoural as observed by the specific staining in the urothelial tumour cells. Moreover, it was also observed staining for L2A5 in areas in which B72.3 did not show reactivity, specifically in low-grade and the invasive front of the tumour T2 (Fig. 7B). Considering the bladder cancer metastasis cases, two (BC6M, BC12M) of the seven cases analysed were positive for both L2A5 and B72.3 mAbs, whereas two cases (BC7M and BC8M) presented staining exclusively with L2A5 (Fig. 7C). In the metastasis cases, L2A5 staining is stronger, mainly observed in tumour cells and absent in the lymphocytic population, vessels or connective tissues (Fig. 7C). Furthermore, enzymatic treatment with sialidase abrogated L2A5 reactivity towards bladder cancer tissues, corroborating its specificity for sialylated targets (Fig. 7D).

**Table 1:** List of bladder cancer cases used in the immunohistochemistry assays.

Patient	Stage	Sample	L2A5		B72.3	
			Extension (%)	Intensity	Extension (%)	Intensity
#1	T2	BC1T	<10	Weak	<10	Weak
#2	T2	BC2T	30	Moderate	30	Moderate
#3	T2	BC3T	40	Strong	35	Strong
#4	T3	BC4T	60	Strong	10	Moderate
#5	T3	BC5T	60	Strong	40	Strong
#6	T3	BC6T	60	Moderate	20	Weak
		BC6M	30	Strong	20	Moderate
#7	T4	BC7T	10	Moderate	10	Weak
		BC7M	<5	Weak	0	Negative
#8	T4	BC8T	70	Strong	20	Weak
		BC8M	5	Weak	0	Negative
#9	T2	BC9M	0	Negative	0	Negative
#10	T3	BC10M	0	Negative	0	Negative
#11	T2	BC11M	0	Negative	0	Negative
#12	T3	BC12M	50	Moderate	30	Weak

Comparison of immunohistochemical staining results of L2A5 and B72.3 mAbs represented as staining intensity and percentage of cells stained.

Note: Tumour stages are defined according to TNM classification. Each bladder cancer sample was numbered BCX. In case of metastasis samples, a suffix M was added.

**Figure 8:**
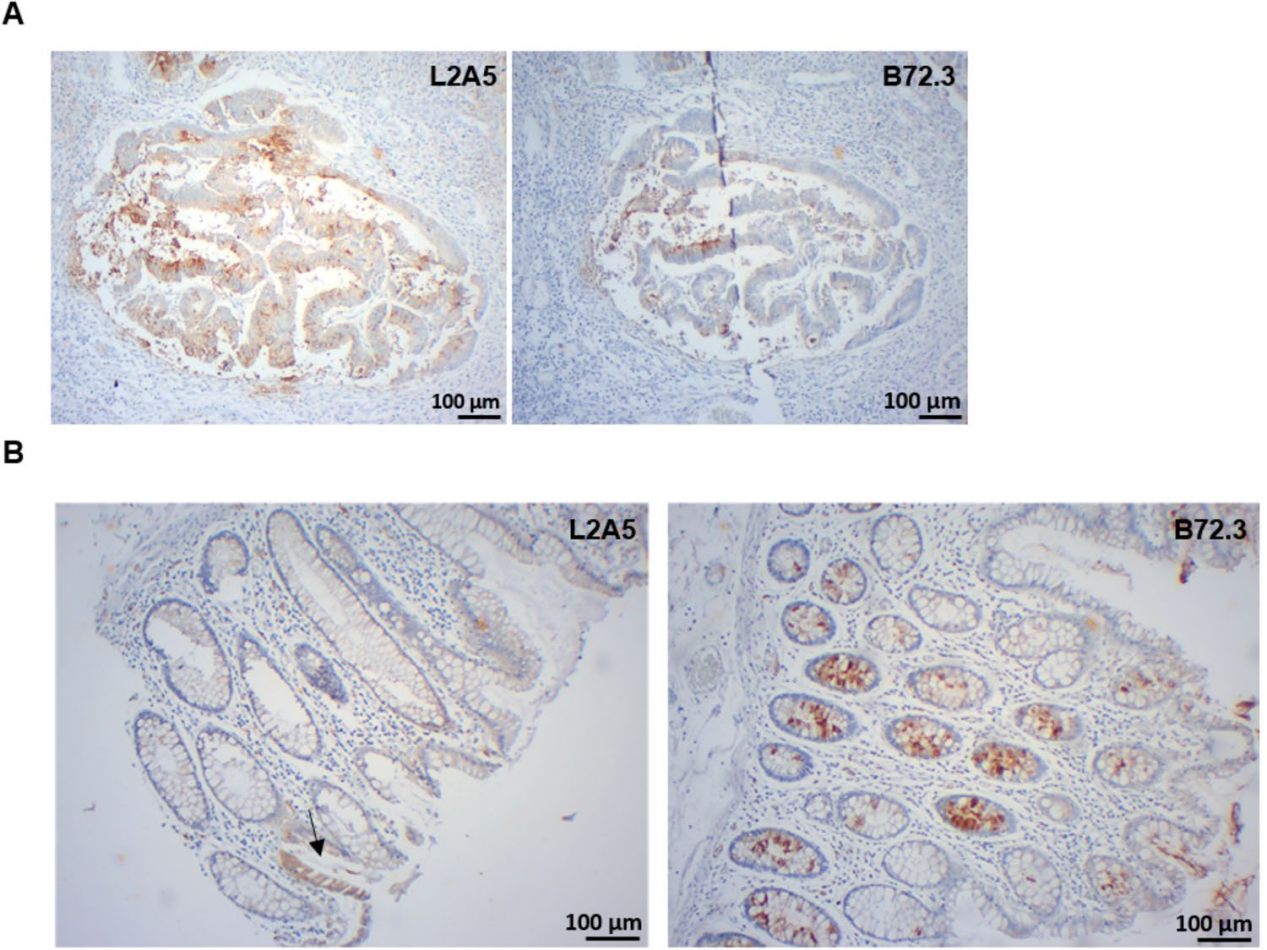
Immunohistochemical evidence of L2A5 mAb staining in colorectal cases and comparison with established anti-STn mAbs. (**A**) Immunohistochemical staining of colorectal cancer tissues. Paraffin embedded tissues were stained with L2A5 and B72.3 mAbs. Images show that L2A5 mAb reactivity shows slightly higher extension and intensity staining compared to B72.3 mAb. (**B**) Immunohistochemical staining of normal colorectal tissues. Paraffin embedded tissues were stained with L2A5 and B72.3 mAbs. Representative images show L2A5 unspecific staining towards enterocytes (arrow) and B72.3 unspecific staining mainly observed in goblet cells.

Regarding colorectal cancer, L2A5 staining showed higher intensity and/or extension (approximately 65% of the cases) in comparison to the B72.3 staining pattern (Fig. 8A), whereas the remaining 35% presented similar staining (Table 2). Yet, one case (CRC4) showed positive reactivity using L2A5 mAb and not with B72.3. In normal colorectal tissues, L2A5 also showed distinct staining patterns regarding signal intensity and extension in comparison to the other antibodies (Fig. 8B). Colorectal mucosa is majorly composed of enterocytes, goblet cells, intraepithelial lymphocytes and extracellular matrix elements. In this mucosa L2A5 presented a faint cytoplasmic reactivity in enterocytes and in some goblet cells, contrasting with the strong staining of B72.3 in goblet cells (Fig. 8B). Moreover, both L2A5 and B72.3 showed no reactivity with intraepithelial lymphocytes, extracellular matrix elements, muscle layer, adipocytes and vessels.

**Table 2:** List of colorectal cancer cases used in the immunohistochemistry assays.

Patient/Sample	Histology/Stage	L2A5		B72.3	
		Extension (%)	Intensity	Extension (%)	Intensity
CRC1	Adenoma	40	Moderate	20	Weak
CRC2	Adenoma	10	Weak	10	Weak
CRC 3	T1	25	Moderate	20	Weak
CRC 4	T1	60	Weak	0	Negative
CRC 5	T2	90	Moderate	90	Moderate
CRC 6	T2	5	Weak	5	Weak
CRC 7	T2	55	Moderate	30	Moderate
CRC 8	T2	90	Moderate	40	Moderate
CRC 9	T3	20	Weak	10	Weak
CRC 10	T3	60	Moderate	50	Moderate
CRC 11	T3	80	Strong	80	Strong
CRC 12	T3	40	Weak	40	Weak
CRC 13	T3	75	Moderate	60	Weak
CRC 14	T4	50	Strong	40	Strong

Comparison of immunohistochemical staining results of L2A5 and B72.3 mAbs represented as staining intensity and percentage of cells stained.

Note: Tumour stages are defined according to TNM classification. Each colorectal cancer sample was numbered CRCX.

**Figure 9:**
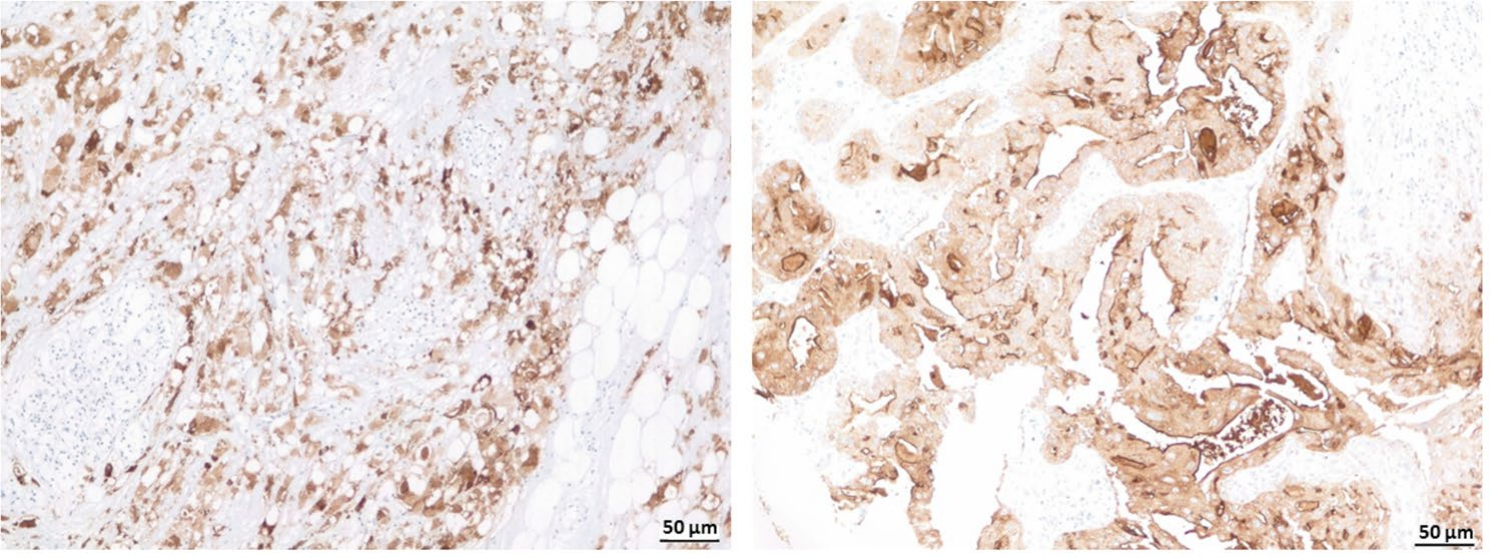
Immunohistochemical reactivity of L2A5 mAb in triple-negative breast cancer. Paraffin embedded triple-negative breast cancer tissues were stained with L2A5 mAb. Image shows two representative cases of immunohistochemical assays.

High-risk cancers comprise not only metastatic cancers, which have aggressive and variable phenotypes, but also cancers with absence of targetable biomarkers such as triple-negative breast cancer (TNBC), which lacks the expression of three cellular receptors: estrogen receptor (ER), progesterone receptor, and the Human Epidermal growth factor Receptor-type 2 (HER2). Personalized medicine for these high-risk cancers could become a reality following new biomarker discovery. In this study, we also investigated the application of the L2A5 mAb in TNBC and we identified a strong and extensive reactivity in 3 cases, out of a total of 15 different cases. The reactivity in two cases was strong and above 50% of the tissue (Fig. 9), while the third case showed a weak staining with nearly 20% of tissue extension. These results confirm the utility of L2A5 mAb to identify TNBC. Overall, our data suggest that the L2A5 mAb specifically stains tumour cells including relevant areas such as metastatic front and low-grade STn regions, in which other mAbs failed to detect.

### Antibody specificity determined by glycan microarrays

**Figure 10:**
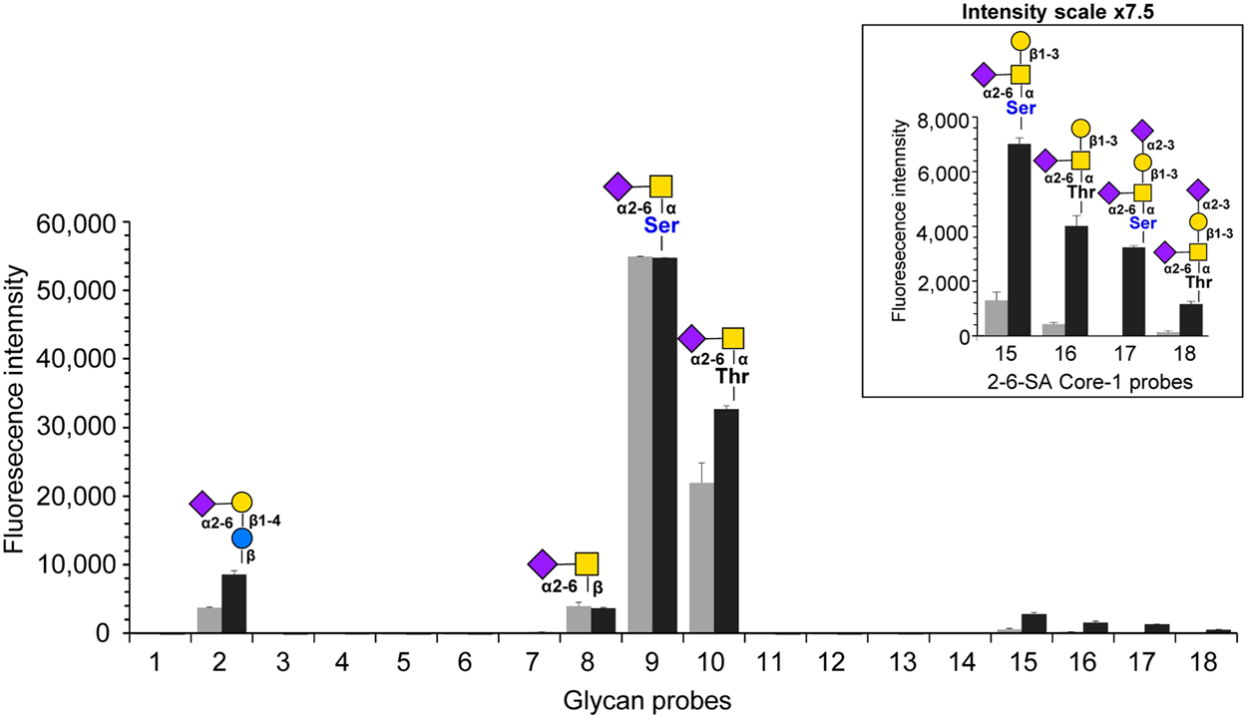
Glycan microarray analysis of anti-STn L2A5 mAb. The L2A5 hybridoma supernatant was diluted 1:2 and the results are shown as the means of fluorescence intensities of duplicate glycan probe spots, printed at 2 and 5 fmol (light and dark grey, respectively). The plot identifies the positive hits obtained. Imaging parameters are PMT 350, laser power 20%. In the inset, the laser scanning conditions were enhanced (PMT 350, laser power 90%) to highlight the binding of L2A5 to the α2–6-linked sialylated core-1 probes. The sequences of sialylated and mucin *O*-glycan core probes in the microarray are listed in Table 3. The monosaccharides are depicted according to Symbol Nomenclature for Graphical Representation of Glycans^65^.

The binding profile detected for L2A5 in initial glycan microarray analysis is highlighted using a subset of neoglycolipid (NGL) probes containing mucin *O*-glycan core sequences; GalNAc core linked to Ser or Thr, and some sialylated oligosaccharide sequences (Fig. 10 and Table 3). This analysis enabled a direct comparison among core *O*-glycan probes that are structurally related to the sialyl-Tn *O*-glycan.

**Table 3:** List of selected NGL probes of mucin *O*-glycan cores and sialylated glycans included in the microarray for assessment of the glycan-binding specificity of L2A5.

Chart ID	Probe	Sequence^*^
**Terminal sialylated**		
1	3′SA-Lac	NeuAcα-3Galβ-4Glcβ-Gly-DA
2	6′SA-Lac	NeuAcα-6Galβ-4Glcβ-Gly-DA
3	LSTb	Galβ-3[NeuAcα-6]GlcNAcβ-3Galβ-4Glcβ-Gly-DA
4	DSLNT	NeuAcα-3Galß-3[NeuAcα-6]GlcNAcß-3Galß-4Glcß-Gly-DA
**Core** ***O*** **-glycan**		
5	GalNAcα-Ser	GalNAcα-O-Ser-DA **Tn-Ser**
6	GalNAcα-Thr	GalNAcα-O-Thr-DA **Tn-Thr**
7	BSM-Di-A1	NeuGcα-6GalNAcβ-AO **Sialyl-Tn (Gc)**
8	BSM-Di-A2	NeuAcα-6GalNAcβ-AO **Sialyl-Tn (Ac)**
9	STn-Ser	NeuAcα-6GalNAcα-Ser-DA **Sialyl-Tn (Ac)-Ser**
10	STn-Thr	NeuAcα-6GalNAcα-Thr-DA **Sialyl-Tn (Ac)-Thr**
11	Core 1-Ser	Galβ-3GalNAcα-Ser-DA **T-Ser**
12	Core 1-Thr	Galβ-3GalNAcα-Thr-DA **T-Thr**
13	SA1(2-3)-Core 1-Ser	NeuAcα-3Galβ-3GalNAcα-Ser-DA **(2-3)-Sialyl-T-Ser**
14	SA1(2-3)-Core 1-Thr	NeuAcα-3Galβ-3GalNAcα-Thr-DA **(2-3)-Sialyl-T-Thr**
15	SA1(2-6)-Core 1-Ser	Galβ-3[NeuAcα-6]GalNAcα-Ser-DA **(2-6)-Sialyl-T-Ser**
16	SA1(2-6)-Core 1-Thr	Galβ-3[NeuAcα-6]GalNAcα-Thr-DA **(2-6)-Sialyl-T-Thr**
17	SA2-Core 1-Ser	NeuAcα-3Galβ-3[NeuAcα-6]GalNAcα-Ser-DA **(2-3,2-6)-diSialyl-T-Ser**
18	SA2-Core 1-Thr	NeuAcα-3Galβ-3[NeuAcα-6]GalNAcα-Thr-DA **(2-3,2-6)-diSialyl-T-Thr**

*The oligosaccharide probes are all neoglycolipids (NGLs). AO: NGLs prepared from reducing oligosaccharides by oxime ligation with aminooxy (AO) functionalized 1,2-dihexadecyl-*sn*-glycero-3-phosphoethanolamine; DA, NGLs prepared using a new aldehyde-terminating lipid reagent to conjugate with amino-terminating glycans. Gly, glycine. This is a subset of a larger array which will be described elsewhere.

The L2A5 tested as a hybridoma serum-free supernatant or after affinity purification exhibited an identical binding profile. A better signal-to-noise ratio was observed with the hybridoma supernatant under the conditions of analysis and the results are shown in Fig. 10. Binding of L2A5 was predominantly observed to the short mucin *O*-glycan core STn disaccharide NeuAcα2–6GalNAc linked to Ser or Thr (probe #9 and #10, respectively). L2A5 could also bind to the STn disaccharide released from bovine submaxillary mucin^41^ and prepared as lipid-linked AO-NGL (probe #8). Under the conditions of this analysis, binding was not detected to the *N*-glycolyl (NeuGc) analogue (probe #7) nor to any of the non-sialylated Tn and core-1 *O*-glycans. Interestingly, the extension of the STn sequence by a Gal residue at the 3-position of the core GalNAc, forming the (α2–6)-sialyl core 1 (probes #15 and #16), or additional α2–3-linked NeuAc at the Gal, forming the (α2–3, α2–6)-di-sialyl core-1 (probe #17 and #18), could be tolerated but the binding was greatly reduced. The weak binding to the α2–6-sialyl lactose (probe 2) is considered as possible cross-reactivity. The probes with sialylation on other backbone-type sequences did not elicit any binding (probes #1, #3 and #4).

In order to identify any cross-reactivity of L2A5 antibody with sialylated glycan sequences or other possible binding epitopes, glycan microarray screening analysis of L2A5 antibody was carried out using a structurally diverse glycan microarray (see Supplementary Fig. S3). The repertoire of sequence-defined lipid-linked glycan probes (see Supplementary Table S1), encompasses a wider range of mammalian type sequences, representative of *N*-glycans (high-mannose-type and neutral and sialylated complex-type), peripheral regions of *O*-glycans; blood group antigen-related sequences on linear or branched backbones and their sialylated and/or sulfated analogs; linear and branched poly-*N*-acetyllactosamine sequences; gangliosides, oligosaccharide fragments of glycosaminoglycans and polysialic acid^42^. Binding to the STn carbohydrate sequence was corroborated in this glycan array. No binding using L2A5 antibody was observed to other types of sialylated sequences found in glycolipids and glycoproteins of mammalian cells, or naturally isolated glycolipids, included in the microarray. An incidental finding was the binding of L2A5 to a synthetic glycosylceramide (GSC-70, probe #467), with the α2–6-sialylated carbohydrate sequence NeuAcα2–6Galβ1–6GalNAcβ1–4Galβ1–4Glcβ^43^. The biological significance of this finding is uncertain, as this type of sequence has not been described to our knowledge on natural glycolipids or *O*-glycoproteins. Notably, the antibody did not bind to the related α2–6-sialylated glycolipids with an internal -Galβ1–3GalNAc- sequence (probes #317 and #318). A weak but detectable binding was also observed to the α2–8-NeuAc-linked disaccharide (probe #350) (prepared as a DH-NGL by reductive amination, which results in ring-opening of the core monosaccharide), but not to the other α2–8-sialylated probes in the array (probes #12, #17, #18, #26, #64, #66, #308-#315, #351-#358). The glycan microarray data confirmed that the L2A5 mAb is specific for the short mucin core STn antigen disaccharide (NeuAcα2–6GalNAcα-Ser/Thr) found in *O*-glycoproteins and has the ability to bind α2–6-linked sialyl core-1, but with much reduced avidity. The other binding signals reported here are interpreted as a possible cross-reaction of the antibody.

## Discussion

STn antigen is a TACA overexpressed in 80% of human carcinomas namely breast, bladder, ovarian and colorectal cancer^23^ but rarely found in fetal and normal adult tissues. Expression of this antigen has been broadly associated with malignant phenotypes and decreased overall survival in patients^7,21,44,45^. STn is a relevant target for tumour immunotherapy and attempts to target this antigen have included both vaccine and antibody-based approaches. An example of such attempts is the vaccine named Theratope developed to treat breast cancer patients, which was composed by synthetic STn epitopes conjugated to keyhole limpet hemocyanin. Theratope reached the clinical trials phase I and II and succeeded in triggering T cell-dependent responses against cancer cells in breast and ovarian cancer patients^46^. However, it failed to demonstrate improved overall survival in phase III clinical trials, probably due to low immunogenicity^47^. As an alternative to vaccines, antibodies targeting STn emerged, leading to the development of mAbs with different specificities against this epitope namely the mAbs B72.3, TKH2, 3F1 and 3P9^33,35,48^. Some of them were tested in the clinic, however, none has been approved for therapeutic purposes and they are mostly used in diagnosis, tumour imaging or radioimmunotherapy^23^. Considering effector functions, so far only the IgM mAb 3P9, reported to specifically recognize STn, was shown to inhibit proliferation and migration of STn-expressing cells as well as on tumour growth by inducing complement dependent cytotoxicity and apoptosis^35^. More recently, Prendergast and colleagues described a subset of engineered antibody-drug conjugates demonstrating *in vitro* efficacy in STn-expressing cell lines and significant tumour growth inhibition in STn-expressing tumour xenograft cancer models^35,36^. Even though these antibodies target the same antigen, different isotypes, binding patterns and functions have been reported. For instance, some anti-STn mAbs preferentially bind to clustered STn while others recognize specific peptide sequences within these clusters^49,50^. Recognizing these limitations and biases, we developed and characterized a novel mAb named L2A5 specifically targeting the STn antigen.

Carbohydrates are known to be challenging targets to raise a strong humoral response^30^. Given this, in the present work, various strategies were devised to produce anti-STn mAbs. Initially and aiming at the development of human anti-STn mAbs, phage display using novel naïve and immune human (derived from breast cancer patients) Fab phage display libraries were screened using biotinylated-STn peptides and MUC1 STn-IgG. Despite the efforts, no specific binders were identified using this strategy possibly due to limitations encountered throughout libraries’ construction and therefore, further improvements are required. We then used the hybridoma technology, and mice immunizations were performed using different immunogens, such as membrane extracts and cell lysates from different cancer cell lines overexpressing STn. However, these immunizations did not induce a robust immune response and therefore STn specific antibodies were not obtained. Only mice immunized with OSM accomplished this aim, leading to the development of a strong immune response and production of anti-STn antibodies. This is in line with previous studies reporting the development of anti-STn mAbs after mice immunization with OSM, as is the case of the mAbs 3F1 and TKH2^34^. Apparently, the STn carrier mucins which are present in OSM elicit higher immunogenicity, in contrast to whole cell protein extracts that contain tolerogenic proteins, such as MUC1 and CEA^51–53^. Notwithstanding, this immunization strategy generated IgM/κ antibodies, typically observed for carbohydrate antigens and most likely due to the documented T cell independent mechanisms induced by TACAs. After screening the potential anti-STn mAbs, mAb L2A5 emerged as a lead candidate. Further characterization using ELISA demonstrated that L2A5 specifically binds to BSM, a STn rich mucin, in a sialic acid-dependent manner. Flow cytometry results further corroborated the sialic acid-dependent binding to surface antigens present in STn-expressing breast and bladder cancer cell lines, previously established^13,53^. In addition, no binding was observed on cancer cells that did not express STn. L2A5 mAb also showed reactivity to membrane extracts from STn^+^ cancer cells and purified MUC1 decorated with STn. These results corroborate once again the specific and dependent binding of L2A5 to STn carrier proteins in different antigen presentation settings, such as STn antigens present on the membrane of different cancer cell lines as well as on purified proteins.

Detection of STn antigens with high specificity and sensitivity in tissue samples is a key aspect in the development of more accurate diagnostics and further application in new targeted therapies. Therefore, cancer specificity of L2A5 mAb was assessed using immunohistochemistry, in which bladder and colorectal cancer tissues were used due to the natural enrichment in mucins and STn epitopes displayed in these cancer models. The results demonstrated the high sensitivity of L2A5 mAb to detect tumour tissue with higher extension, in a sialic acid-dependent binging, compared to the widely used anti-STn antibodies. L2A5 reacts with tissue from low-grade and invasive fronts of tumours, which are not detected by other antibodies. This high sensitivity of L2A5 mAb to detect tumour tissue is probably due to its broader specificity to tumour antigens, since it reacts with STn antigen but also with other TACAs containing sialic acid, as shown by the glycan microarray data. Nevertheless, at this stage it remained unclear whether the L2A5 higher sensitivity to stain tumour tissue was due to the detection of low density STn antigen not reachable by other mAbs or due to additional specificity to other sialic acid containing TACAs.

The assignment of binding specificity to oligosaccharide sequences is a key feature to assess in a newly developed anti-carbohydrate antibody. To this end, the L2A5 mAb was analysed using glycan microarrays confirming the predicted binding to the disaccharide STn antigen, but unexpectedly also to α2–6-linked sialyl core-1 probes, albeit with much reduced avidity. To our knowledge, this is the first report of an antibody able to bind (2–6)-sialyl core-1 structures.

Glycan microarrays analysis has revealed different glycan binding patterns for other available anti-STn mAbs, despite the fact that they predictably target the same antigen^54^. For instance, using STn-glycopeptide microarrays, the mAbs TKH2 and 3F1 showed binding to STn but with a preference for clustered di-STn-glycosylated peptide sequences^49,50^. The widely used mAb B72.3 showed a more selective reactivity to a specific peptide structure within the clustered bis-STn-epitopes^55^. However, this antibody also showed binding to multiple STn-related oligosaccharides, including the Tn antigen (GalNAc)^36^. Conversely, the novel L2A5 mAb herein described demonstrates a high specific binding to cancer-related short or truncated mucin-type STn and α2–6-linked sialyl core-1 sequences, overexpressed in several cancers and involved in cancer progression and metastasis. Antibodies with such specificity patterns are particularly interesting due to their high tumour specificity and low or absent reactivity to normal cells, endorsing their application as a new diagnostic tool as well as in antibody-based anti-cancer therapies. Additionally, these antigens are recognized by a number of immune receptors such as the sialic acid binding proteins (Siglecs) which may contribute to immune tumour evasion^56,57^. Correlating with the results obtained using immunohistochemistry, this restricted and different specificity may explain the differential tissue staining pattern when compared with other anti-STn mAbs, such as B72.3.

In addition, an IgM antibody named 3P9 was developed showing inhibitory effects in cell proliferation and migration as well as apoptosis of STn-expressing tumour cells *in vivo* and *in vitro*^35^. Despite the detailed characterization and similarities with our antibody regarding antibody isotype, glycan specificity of 3P9 mAb remains elusive to sialylated oligosaccharides^50^.

In summary, we developed and characterized a novel anti-STn mAb using a wide range of assays proving the fine specificity of this antibody towards the STn antigen and sialylated cancer biomarkers. Therefore, our antibody can serve not only for diagnostic purposes, but also effective targeting and blocking of cancer-associated antigens involved in mechanisms underlying tumour progression in multiple types of cancer. Furthermore, the potential application of L2A5 as antibody-drug conjugates or chimeric antigen receptors could be considered effective therapeutic approaches.

## Materials and Methods

### Ethics statement

This project followed the social, ethical and environmental laws or principles accepted in Portugal and in the European Union. Experiments were conducted by people certified by the Portuguese national body for experimental animal manipulations “Direcção Geral de Alimentação e Veterinária” (DGAV). The facilities at IHMT/UNL animal room, procedures for maintenance and care of animals as well the experimental scheme used (immunization, blood collection, spleen removal and euthanasia procedures) are accredited by the Portuguese DGAV and in accordance with relevant guidelines and regulations of the ethical committee of IHMT/UNL.

### Cell lines and cell culture

The human breast cancer cell line MDA-MB-231 wild-type (WT; ATCC®HTB-26™) and the transfectant variant (MDA-MB-231 STn^+^) generated to overexpress the human ST6GalNAc-I were kindly provided by professor Philippe Delannoy from Université de Lille UGSF, Villeneuve d’Ascq, France^53^. Transduced variants of bladder cancer cell line MCR were generated to overexpress the cDNA of the human ST6GalNAc-I (MCR STn^+^ cell line) or mock transduced (MCR NC cell line) as previously described^13^. Both MDA-MB-231 WT and MCR NC do not express STn and therefore, are herein referred as MDA-MB-231 STn^−^ and MCR STn^−^ cells, respectively. For all experiments, cells were harvested from culture flasks using Trypsin-EDTA (Gibco, 25200056) in sterile Phosphate-Buffered Saline (PBS). All cell lines were cultured in Dulbecco’s Modified Eagle Medium (DMEM) (Gibco, 11965-084), supplemented with 10% (v/v) Fetal Bovine Serum (FBS, Sigma, F7524), 2 mM L-glutamine (Gibco, 25030081), 100 U/ml of penicillin (Gibco, 15140122) and 100 µg/ml of streptomycin (Sigma, S9137) at 37 °C in a 95% humidified atmosphere containing 5% CO_2_. Mycoplasma tests were performed routinely to ensure that the cell lines were free from mycoplasma contaminations. For this, a polymerase chain reaction (PCR)-based detection method was performed using specific primers, which specifically amplify the intergenic spacer region between the 16S and the 23S rRNA genes of mycoplasmas, as described by Harasawa, R. *et al*.^58^.

### Antibodies and reagents

The anti-STn mAbs B72.3, and 3F1^33,34^ were kindly provided by Professor Celso Reis (Porto University, Portugal). The mAb L2A5 was generated by our laboratory as herein described. The secondary antibodies react with mouse immunoglobulins of all classes, namely the rabbit anti-mouse Immunoglobulins (Ig) conjugated with fluorescein isothiocyanate (FITC) (Dako, F0232) and goat anti-mouse Ig conjugated with horseradish peroxidase (HRP) (BD Biosciences, 554002). Neuraminidase (sialidase) from *Clostridium perfringens* was purchased from Roche Diagnostics (Roche, 11585886001). Asialo-BSM was prepared by acid desialylation of BSM and kindly provided by Professor Fabio Dall’Olio (Università di Bologna, Italy).

### Animals

Female Balb/c mice aged 6 weeks (Harlan UK) were used and fed *ad libitum*. Good animal handling practices, as well as good laboratory practices used at IHMT/UNL animal room are according EU Directives (2000/54/CE Exposure to biological agents, 88/320/CE Good laboratory practices, 90/679/CE, 405/98/PT and 1036/98/PT). Efforts were made to minimize suffering, and at the completion of the study, the mice were euthanized by CO_2_ narcosis followed by cervical dislocation.

### Development of anti-STn mAbs

MAb production was performed according to the hybridoma technology described by Köhler and colleagues^38^. Six-week-old female Balb/c mice (Harlan UK) were immunized intraperitoneally with 10 µg of ovine submaxillary mucin (OSM, Accurate Chemical & Scientific Corp) emulsified 1:1 (v/v) with complete Freund’s adjuvant (Sigma, F5881) followed by 2 additional injections of OSM emulsified with incomplete Freund’s adjuvant (Sigma, F5506) with intervals of 21 days. Blood samples were collected from the mice cheek throughout the immunization procedure and collected serum was screened by indirect Enzyme-Linked Immunosorbent Assay (ELISA), as described below. When the serum presented the desired and specific immune response, a final boost injection was performed three days before the animals were euthanized and subsequently the spleens were harvested. Splenocytes from the immunized mouse were mixed with Sp2/0-Ag14 myeloma cells (ATCC® CRL-1581™) at a ratio of 3:1 and fused in the presence of polyethylene glycol/dimethylsulphoxide (PEG/DMSO, Hybri-max Sigma-Aldrich, P7306) using a standard protocol. Cells were plated into 96-well flat bottom micro plates (Orange Scientific) and maintained in selection medium, RPMI medium (Gibco, 42401-018) supplemented with HAT medium (1 × 10^−4^ M hypoxanthine, 4 × 10^−7^ M aminopterin, 1.6 × 10^−5^ M thymidine (Sigma, H0262)), 10% (v/v) FBS (Sigma, F7524), 2 mM L-glutamine, 0.2 mg/ml gentamycin (Sigma, G1397), 1 mM sodium pyruvate (Gibco, 11360-039), 1% (v/v) non-essential amino acids (Gibco, 11450-050) and incubated at 37 °C, 95% humidity containing 5% CO_2_ for 7–12 days. Hybridoma cells were expanded in HAT medium, screened by indirect ELISA and cloned by the limited dilution method at least three times to obtain stable single clone cell lines. Selected hybridomas were further adapted to grow in medium without HAT supplementation. One hybridoma termed L2A5 demonstrating specific binding to sialylated structures, particularly STn, was selected and further used in several experiments.

### Hybridoma screening and antibody specificity using ELISA

Mouse serum titrations and screening of hybridoma supernatants were determined by indirect ELISA against Bovine Submaxillary Mucin (BSM), a STn-expressing protein. Additionally, BSM deprived of STn antigens (asialo-BSM) was used as negative control. 96-well polystyrene plates (Corning) were coated with 50 µl of BSM (3 µg/ml) dissolved in PBS pH = 7.2 and incubated overnight at 4 °C. In order to assess the sialic acid-dependent binding of screened hybridoma supernatants to sialylated structures, 25 mU/ml of sialidase in 10 mM Na_2_HPO_4_ in Milli-Q water (pH = 6.0) was added to the BSM coated wells and incubated for 90 min at 37 °C. After sialidase treatment, the plates were washed three times with PBS containing 0.05% (v/v) Tween 20 (PBS-T) followed by blocking with 5% skim milk powder in PBS-T for 60 min. Following washing steps, diluted mouse sera or hybridoma supernatants were added to the wells and incubated for 90 min. Further washing steps were performed followed by incubation with HRP-conjugated goat anti-mouse Ig (1:1000) for 60 min. After three additional washing steps, 50 µl of 3,3′,5,5′-tetramethylbenzidine (TMB) (Thermo Scientific, 002023) substrate were added to each well, plates were incubated in the dark and the reaction was stopped by adding 50 µl of 1 M HCl. Optical Density (OD) was measured at 450 nm on a microplate reader (SpectraMax 190 Microplate Reader, Molecular Devices). The mouse producing the highest titer of antibodies of interest was selected for fusion. To screen the antibody production of hybridoma cells, the same procedure was implemented on the hybridoma cell culture supernatants. Unless otherwise mentioned, the incubation steps were performed at room temperature.

### Antibody isotyping ELISA

The isotype of the newly developed mAb was determined using isotype capture ELISA and IsoStrip™ Mouse Monoclonal Antibody Isotyping Kit (Roche, 11493027001) according to manufacturer’s instructions. Briefly, goat anti-mouse IgG1, IgG2a, IgG2b, IgG3, IgM and IgA (Sigma) diluted 1:1000 in PBS were adsorbed in the wells of an ELISA plate (Nunc) for 60 min at 37 °C. The plate was washed three times with PBS-T and hybridoma supernatants were added and incubated for 60 min. After washing, appropriate dilution of HRP-conjugated anti-mouse Ig (Fab specific) (Sigma) was added for 30 min. Following three washing steps, the reaction was detected using TMB substrate, stopped using HCl and the OD measured as described above.

### Antibody purification

For antibody purification, L2A5 hybridoma cells were cultured in serum-free medium (Hybridoma-SFM, Thermo Scientific, 12045076). Hybridoma supernatant containing L2A5 mAbs was recovered and centrifuged at 1000 *g* for 10 min. The supernatant was collected and diluted twofold in PBS. Considering the composition of the hybridoma-SFM medium used, low level of contaminants was present in the recovered supernatant and therefore a desalting strategy was considered useful to recover and purify the anti-STn IgM antibodies. For that, the supernatant was loaded onto desalting columns (HiPrep 26/10 Desalting, GE Healthcare Life Sciences, 45-000-266) and dialyzed against PBS (pH 7.2). Due to the high volume of supernatant available, four desalting columns were connected and used in series. The protein peak was registered by spectrophotometry measuring the OD at 280 nm. Eluted fractions containing antibodies were further concentrated using a 200 kDa MW cut-off concentrator system (Vivaflow® 200, Sartorius). A final concentration step to reduce the final sample volume was performed by ultrafiltration using a 100 kDa MW cut-off filter (Amicon, Millipore). Antibody concentration was assessed using the Pierce™ modified Lowry protein assay kit (Thermo Scientific, 23240)^59^.

### Antiboby binding kinetics using bio-layer Interferometry

Affinity of the L2A5 mAb (K_D_ value) was determined by bio-layer interferometry, using OCTET RED96 system (Pall ForteBio). A biotinylated peptide derived from MUC1 containing STn antigens (kindly provided by Professor Hans Wandall, University of Copenhagen, Denmark) was immobilized on streptavidin biosensor tips. Antigen was coated at a concentration of 10 µg/ml and rate constants for association and dissociation were measured using L2A5 concentrations ranging from 3.5 to 40 nM diluted in PBS. All sensorgrams were referenced for buffer effects and fitted using the OctetRED user software (Pall ForteBio). The kinetic parameters of association (K_on_) and dissociation (K_off_) constants were determined and further used to calculate the equilibrium dissociation constant (K_D_).

### Flow cytometry analysis

Cell surface binding of anti-STn antibodies or hybridoma supernatants was determined by flow cytometry using non-STn-expressing parental cells and stable human bladder and breast cell lines expressing STn. Approximately 3 × 10^5^ cells were harvested per condition and resuspended in PBS buffer. To assess the sialic acid-dependent binding of screened hybridoma supernatants and antibodies, cells were treated with sialidase at 100 mU/ml diluted in sialidase buffer for 90 min at 37 °C, 5% CO_2_^40^. After sialidase treatment, cells were washed and incubated for 30 min at 4 °C with anti-STn mAbs B72.3 or 3F1, and hybridoma supernatants diluted in PBS. Subsequent washing steps were performed, and primary antibodies were detected with FITC conjugated anti-mouse Ig (Dako, F0232; dilution 1:10) for 15 min in the dark. After washing, data acquisition was carried out using Attune Acoustic Focusing Cytometer (Applied Biosystems) in which 10 000 events, gated on forward scatter (FSC) vs. side scatter (SSC), were collected for each sample. Data was subsequently analysed using FlowJo vX 0.7 software.

### Western blot analysis

Membrane proteins were isolated from non-treated or sialidase treated cell lines_,_ using Mem-PER™ Plus Membrane Protein Extraction Kit (Thermo Scientific, 89842) according to the manufacturer’s instructions. Desialylation was performed using 100 mU/ml of sialidase diluted in sialidase buffer for 90 min at 37 °C, 5% CO_2_. The amount of protein obtained was estimated using Pierce™ BCA Protein Assay Kit (Thermo Scientific, 23225) following manufacturer’s recommendations. Membrane protein extracts (50 µg) or purified proteins containing or not STn (1 µg) - MUC1 STn-IgG and unglycosylated MUC1 STn-IgG (kindly provided by Professor Joy Burchell, King’s College London^60^) – were denatured and loaded onto 8% gradient acrylamide gel, submitted to SDS–PAGE electrophoresis under reducing conditions and electrophoretically transferred onto polyvinylidene difluoride (PVDF) membranes (Amersham Hybond P 0.2 µm PVDF, GE Healthcare Life Sciences) in accordance with standard procedures. Membranes were blocked with 10% skim milk powder in TBS Tween 0.1% (TBS-T) for 1 h followed by incubation with primary antibodies anti-STn 3F1 or L2A5 supernatant diluted in TBS-T overnight at 4 °C. After washing with TBS-T, labelled proteins were detected using HRP conjugated goat anti-mouse Ig diluted 1:2500 in TBS-T for 1 h. Three additional washing steps were performed, labelled proteins were detected using Lumi-Light Western Blotting Substrate (Roche, 12015200001) and further exposed to an X-ray film (Amersham Hyperfilm ECL, GE Healthcare, 28906836).

### Immunohistochemistry analysis

A series of 29 clinical cases comprising 15 bladder tumours (eight muscle-invasive bladder cancer (MIBC) and seven ganglionar metastasis) and 14 colorectal tumours (adenocarcinomas and adenomas) were obtained from the Portuguese Institute for Oncology of Porto (IPO-Porto, Portugal), according to the local committee of ethics. In addition, 5 cases of tumour-adjacent normal colorectal tissue and 15 triple negative breast cancer tissues were also included. All procedures involving tumour tissues have been authorized by the Portuguese Institute of Oncology of Porto ethics committee (ref. CES 162/013 for colorectal tumours; CES 87/017 for bladder cancer tumours). Formalin-fixed, paraffin embedded (FFPE) tissues were screened by immunohistochemistry using the biotin/streptavidin system. Briefly, FFPE tissue sections were deparaffinized with xylene, rehydrated with a graded series of alcohol washes and subjected to heat-induced antigen retrieval using citrate buffer pH = 6.0 (Vector Laboratories) for 15 min in the microwave, after pre-heating of the solution at maximum power rating for 5 min. Sections were incubated with 0.3% hydrogen peroxide (Merck KGaA) for 25 min, blocked with UV Block® (Thermo Scientific, TL-060-HD) and incubated overnight at 4 °C in a wet chamber with anti-STn mAbs B72.3, and L2A5 diluted 1:5; and 1:3 in 5% BSA-PBS, respectively. After washing with PBS-Tween, biotinylated secondary antibody anti-mouse IgG (H + L) was added to tissue sections, before incubation with streptavidin (Thermo Scientific, 434301). Antibodies’ binding was detected by incubation with 3,3′-diaminobenzidine (ImmPACT™ DAB, Vector Laboratories, SK-4105) for 4 min. Nuclei were counterstained with hematoxylin for 1 min. Positive and negative control sections were tested in parallel. The negative control sections were performed devoid of primary antibody. Positive controls consisted of known positive tumour tissues for the antigen in study. Tumours were classified as positive when mAb immunoreactivity was observed by microscopic presence of brown chromogenic product in tumour cells. Antibodies’ staining was assessed double-blindly by two independent observers and validated by an experienced pathologist. Whenever there was a disagreement, the slides were reviewed, and consensus was reached. To evaluate sialic acid-dependent binding of the antibodies, tissues were treated with sialidase after incubation with hydrogen peroxide, as above mentioned.

### Glycan microarray analyses

Glycan microarray analyses were performed using neoglycolipid (NGL)-based microarray platforms. Details of the glycan probes, generation of the microarrays, imaging and data analysis are available in the supplementary glycan microarray document (see Supplementary Table S2) in accordance with the MIRAGE (Minimum Information Required for A Glycomics Experiment) guidelines for reporting glycan microarray-based data^61^. The sialylated and mucin *O*-glycan core probes used for the initial analysis of the L2A5 antibody were printed in a newly developed microarray comprising sequence-defined NGL probes from synthetic amino-terminating sugars and naturally-derived glyco-amino acids derivatized to an aldehyde-terminating lipid (in house designation “DA-NGL microarray”; preparation of the probes and the full microarray comprising probes with other glycan sequences, such as glucans and neutral *N*-glycans, which did not show binding with L2A5 antibody, will be published elsewhere). The microarray of sequence-defined lipid-linked glycan probes used for screening analysis of L2A5 antibody (see Supplementary Table S1) consist of 475 probes, as described in Palma *et al*.^42^. These are a sub-set of a recently generated large screening microarray containing around 900 glycan probes (in-house designation “Array Sets 42–56”, which will be published elsewhere). For construction of the microarrays, the NGL probes were immobilized non-covalently at 2 and 5 fmol/spot onto 16-pad nitrocellulose-coated glass slides, as previously described^62^. The microarrays were probed with the anti-STn mAb preparations, following described protocols^63^. In brief, nonspecific binding sites were blocked using the blocking solution casein (Pierce, 37528) diluted to 0.02% (v/v) in PBS with addition of 1% BSA. The microarray slides were overlaid for 90 min with L2A5 hybridoma supernatant (1:2 dilution) or purified antibody (10 µg/ml) diluted in the blocking solution. The binding was detected using biotinylated anti-mouse IgM (Vector BA-2020, diluted 1:300) followed by Alexa Fluor-647-labeled streptavidin at 1 μg/ml (Molecular Probes, S21374) diluted in the blocking solution. After fluorescence quantitation, microarray data analysis was performed using a suitable software developed by Mark Stoll from the Glycosciences Laboratory, as previously described^64^.

### Data availability

The datasets generated during and/or analysed during the current study are available from the corresponding author on reasonable request.

## Electronic supplementary material

Supplementary data
