# Supplementary material for: Novel monoclonal antibody L2A5 specifically targeting sialyl-Tn and short glycans terminated by alpha-2–6 sialic acids
Source: Sci Rep. 2018 Aug 15;8:12196. doi: 10.1038/s41598-018-30421-w (PMC6093877; doi:10.1038/s41598-018-30421-w)
Supplement: Supplementary file 1 — Supplementary data [file 41598_2018_30421_MOESM1_ESM.docx]

**Supplementary Information**

**Novel monoclonal antibody L2A5 specifically targeting sialyl-Tn and short glycans terminated by alpha-2-6 sialic acids**

Liliana R. Loureiro**^1,2^**, Diana P. Sousa**^1^**, Dylan Ferreira**^3^**, Wengang Chai**^4^**, Luís Lima**^3,5,6^**, Carina Pereira**^7,8^**, Carla B. Lopes**^9^**, Viviana G. Correia**^10^**, Lisete M. Silva**^4^,** Chunxia Li**^11^**, Lúcio Lara Santos**^3,12,13^**, José Alexandre Ferreira**^3,6,8,12,14^**, Ana Barbas**^2,15^**, Angelina S. Palma**^4,10^**, Carlos Novo**^1,16^*** and Paula A. Videira**^1,17^***

**
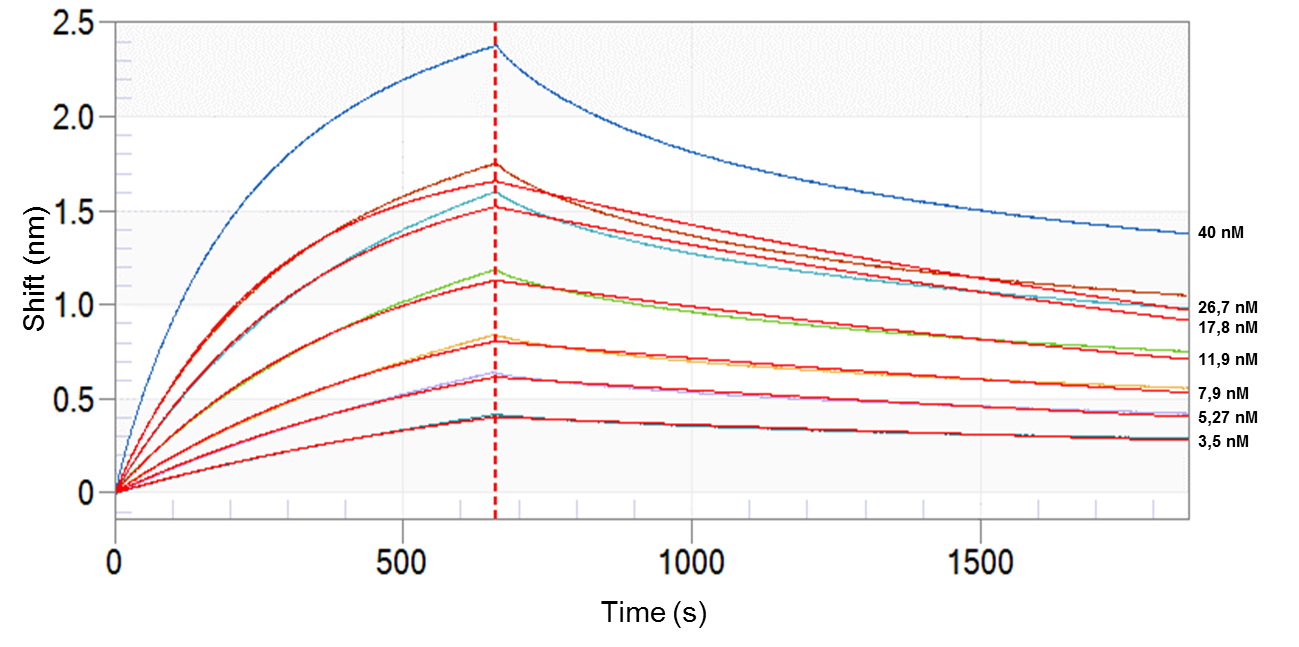
**

| **mAb** | **K_on_ (M^-1^ s^-1^)** | **K_off_ (s^-1^)** | **K_D_ (nM)** |
| --- | --- | --- | --- |
| L2A5 | 1,80×10^5^ | 3,76×10^-4^ | **2,25** |

**Supplementary Figure S1 – L2A5 mAb binding kinetics to immobilized MUC1 STn determined using bio-layer interferometry.** L2A5-MUC1 STn association and dissociation curves (demarcated by a vertical dashed red line) were obtained through serial dilutions of L2A5 mAb (3.5 nM-40 nM) plus buffer controls using Octet Red 96 and Octet acquisition software. Raw data and the respective nonlinear regression fitting curves (red curves) are shown and labelled accordingly. No fitting curve was obtained using 40 nM of L2A5 mAb due to the high OD shift values obtained. Calculated K_D_, K_on_ and K_off_ values are shown in a tabular form.

| 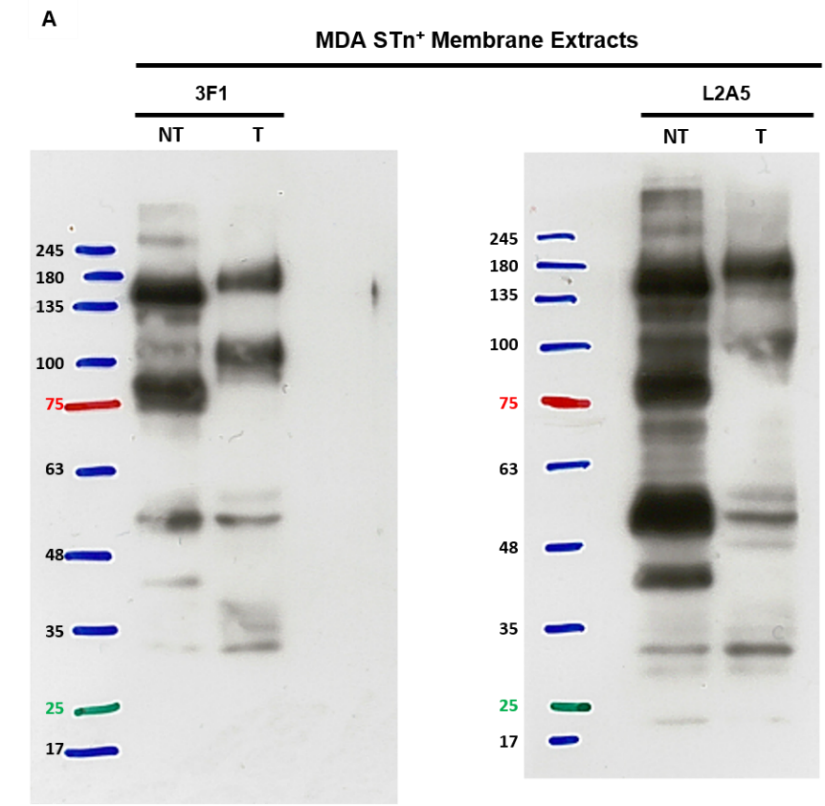 | |
| --- | --- |
| 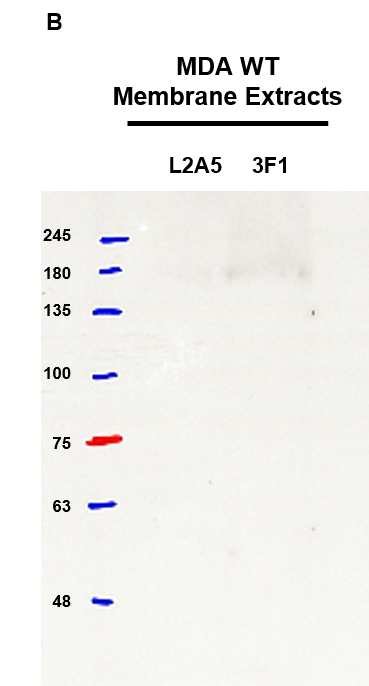 | 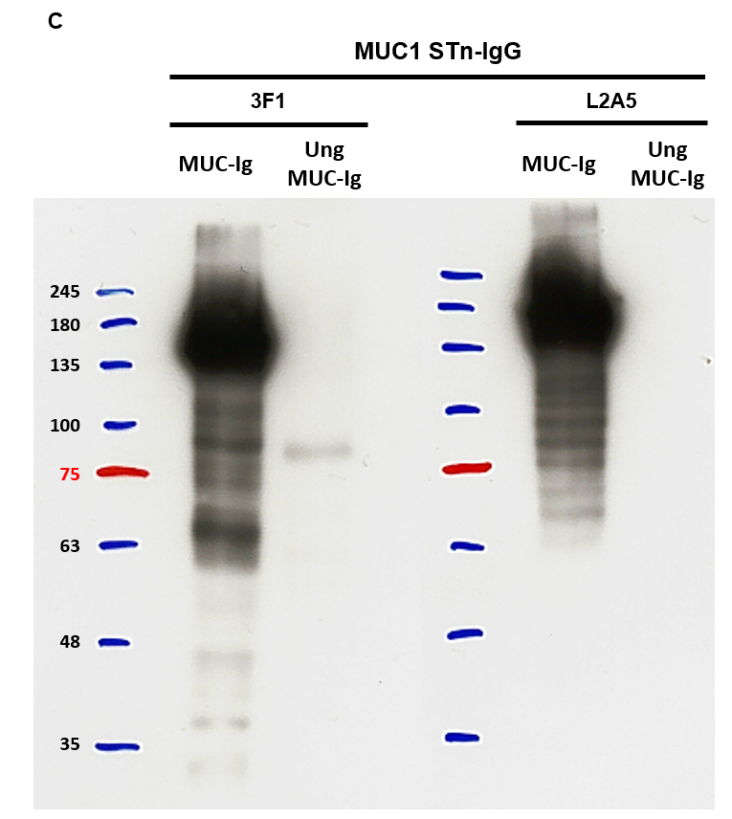 |

**Supplementary Figure S2 –** **Full length western blot of anti-STn mAb staining using STn-expressing cell lines and MUC1 STn – IgG (from Fig.6).** MDA-MB-231 STn^+^ membrane extracts (**A**), MDA-MB-231 WT membrane extracts (**B**) and chimeric protein MUC1 STn-IgG (**C**) were stained with L2A5 and 3F1 (control) mAbs. Blotting was performed using untreated membrane extracts (NT) and chimeric protein (MUC-Ig) as well as membrane extracts samples that were desialylated using sialidase (T) or unglycosylated MUC1 STn-IgG protein (Ung MUC-Ig).


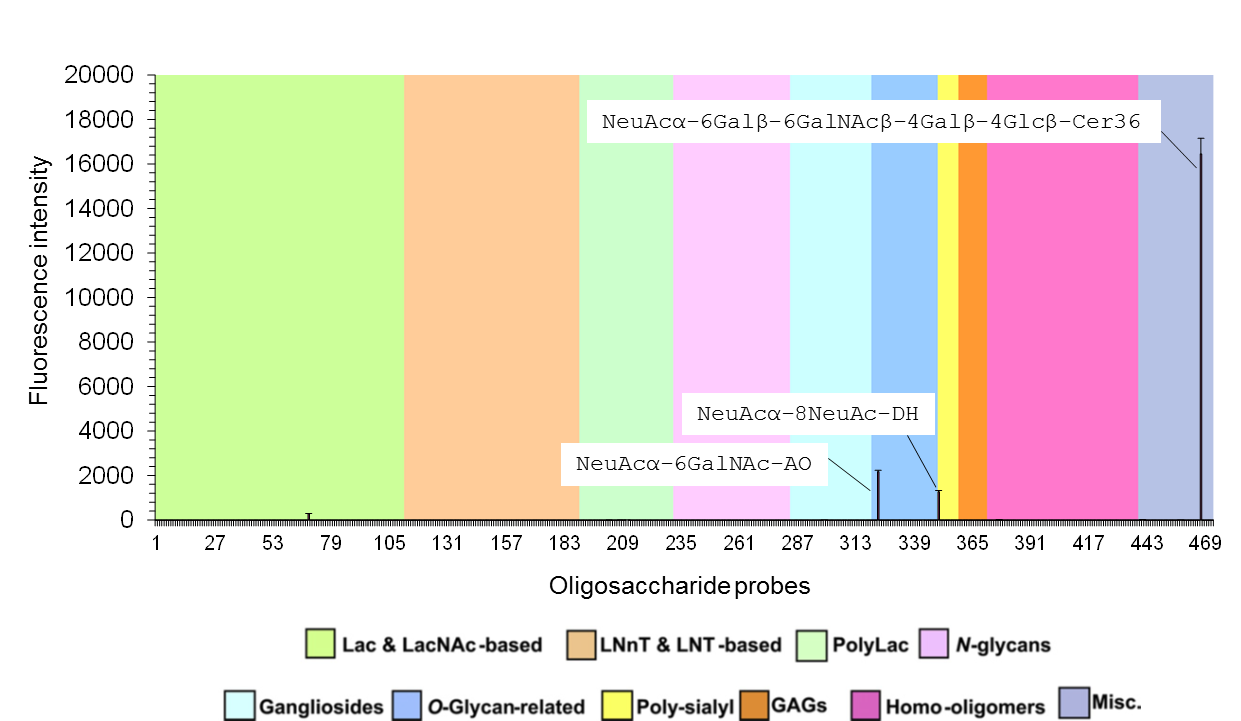


**Supplementary Figure S3 - Glycan microarray screening analysis of anti-STn L2A5 mAb.** The L2A5 hybridoma supernatant was tested at 1:2 dilution. The results are the means of fluorescence intensities of duplicate spots, printed at 5 fmol. The error bars represent half of the difference between the two values. The probes are grouped according to their backbone-type sequences as annotated by the colored panels: disaccharide based: lactose (Lac) and *N*-acetyllactosamine (LacNAc); tetrasaccharide based: lacto-*N*-neo-tetraose (LNnT) and lacto-*N*-tetraose (LNT); poly-N-acetyllactosamine (PolyLac); *N*-glycans; gangliosides; *O*-glycan-related; polysialyl; glycosaminoglycans (GAGs); homo-oligomers of glucose and of other monosaccharides, and other non-classified sequences (miscellaneous, Misc). The list of probes and their sequences are in Supplementary Table S1.

**Supplementary Table S1 -** List of glycan probes, their sequences and the fluorescence binding intensities elicited with L2A5 mAb.

| **Pos^a^** | **Probe^b^** | **Sequence** | **L2A5  mAb Binding^c^** | **Error^d^** |
| --- | --- | --- | --- | --- |
| ***Lactose and N-acetyllactosamine-based*** | | | | |
| 1 | Galactocerebrosides | Galβ-Cer | - | - |
| 2 | H-Di | Fucα-2Gal-DH | - | - |
| 3 | A-Tri | GalNAcα-3Gal-DH  │  Fucα-2 | - | - |
| 4 | B-Tri-AO | Galα-3Gal-AO  │  Fucα-2 | - | - |
| 5 | GSC-426 | (3-deoxy,3-carboxymethyl)Galβ-C30 | - | - |
| 6 | Sulfatide | SU-3Galβ-Cer | - | - |
| 7 | GSF-1 | SU-3Galβ-C30 | - | - |
| 8 | GSC-209 | GlcAβ-3Galβ-Cer42 | - | - |
| 9 | GSC-210 | SU-3GlcAβ-3Galβ-Cer42 | - | - |
| 10 | GSC-187 | NeuAcα-3Galβ-C29 | - | - |
| 11 | GSC-40 | NeuAcα-(S)-3Galβ-Cer42 | - | - |
| 12 | GSC-230 | NeuAcα-8NeuAcα-3Galβ-Cer36 | - | - |
| 13 | GSC-27 | NeuAcα-6Galβ-Cer36 | - | - |
| 14 | GSC-144 | KDNα-6Galβ-Cer36 | - | - |
| 15 | GSC-13 | NeuAcα-(S)-6Galβ-Cer36 | - | - |
| 16 | GSC-72 | NeuAcα-(S)-6Galβ-(S)-Cer36 | - | - |
| 17 | GSC-231 | NeuAcα-8NeuAcα-6Galβ-Cer36 | - | - |
| 18 | GSC-439 | NeuAcα-8NeuAcα-8NeuAcα-6Galβ-Cer36 | - | - |
| 19 | Glucocerebrosides | Glcβ-Cer | - | - |
| 20 | GSF-19 | SU-6Glcβ-C30 | - | - |
| 21 | GSC-60 | NeuAcα-6Glcβ-Cer36 | - | - |
| 22 | GSC-9 | NeuAcα-(S)-6Glcβ-Cer36 | - | - |
| 23 | GSC-62 | NeuAcα-2Glcβ-Cer36 | - | - |
| 24 | GSC-59 | NeuAcα-6GlcNAcβ-Cer36 | - | - |
| 25 | GSC-95 | NeuAcα-(S)-6GlcNAcβ-Cer36 | - | - |
| 26 | GSC-232 | NeuAcα-8NeuAcα-6Glcβ-Cer36 | - | - |
| 27 | Lactocerebrosides | Galβ-4Glcβ-Cer | - | - |
| 28 | Lac | Galβ-4Glc-DH | - | - |
| 29 | Lac-AO | Galβ-4Glc-AO | - | - |
| 30 | GSC-432 | (3-deoxy,3-carboxymethyl)Galβ-4Glcβ-C30 | - | - |
| 31 | GSC-296 | GlcAβ-3Galβ-4Glcβ-C30 | - | - |
| 32 | GSC-353 | SU-3GlcAβ-3Galβ-4Glcβ-C30 | - | - |
| 33 | GalNAcα-3Galβ-4Glc | GalNAcα-3Galβ-4Glc-DH | - | - |
| 34 | Globotri-AO | Galα-4Galβ-4Glc-AO | - | - |
| 35 | Ceramide trihexoside | Galα-4Galβ-4Glcβ-Cer | - | - |
| 36 | Globoside (P-antigen) | GalNAcβ-3Galα-4Galβ-4Glcβ-Cer | - | - |
| 37 | Forssmann glycolipid | GalNAcα-3GalNAcβ-3Galα-4Galβ-4Glcβ-Cer | - | - |
| 38 | Fuc(3)-Lac-AO | Galβ-4Glc-AO  │  Fucα-3 | - | - |
| 39 | GSC-430 | (3-deoxy,3-carboxymethyl)Galβ-3Glcβ-C30  │  Fucα-4 | - | - |
| 40 | GSC-260 | 3-deoxy,3-carboxymethyl-Galβ-4Glcβ-C30  │  Fucα-3 | - | - |
| 41 | GSC-150 | SU-3Galβ-4Glcβ-C30  │  Fucα-3 | - | - |
| 42 | GSC-160 | SU-3Galβ-4Glcβ-Cer36  │  Fucα-3 | - | - |
| 43 | NeuAcα-(3')Lac | NeuAcα-3Galβ-4Glc-DH | - | - |
| 44 | NeuAcα-(3')Lac-AO | NeuAcα-3Galβ-4Glc-AO | - | - |
| 45 | Neu4,5Ac-(3')Lac | (4-OAc)NeuAcα-3Galβ-4Glc-DH | - | - |
| 46 | Neu4,5Ac-(3')Lac-AO | (4-OAc)NeuAcα-3Galβ-4Glc-AO | - | - |
| 47 | GSC-16 | NeuAcα-3Galβ-4Glcβ-Cer32 | - | - |
| 48 | GSC-178 | NeuAcα-3Galβ-4Glcβ-Cer34 | - | - |
| 49 | GSC-17 | NeuAcα-3Galβ-4Glcβ-Cer36 | - | - |
| 50 | GSC-18 | NeuAcα-3Galβ-4Glcβ-Cer42 | - | - |
| 51 | GSC-197 | KDNα-3Galβ-4Glcβ-Cer28 | - | - |
| 52 | GSC-199 | KDNα-3Galβ-4Glcβ-C30 | - | - |
| 53 | GSC-198 | KDNα-3Galβ-4Glcβ-Cer34 | - | - |
| 54 | GSC-75 | (4-deoxy)NeuAcα-3Galβ-4Glcβ-Cer36 | - | - |
| 55 | GSC-76 | (7-deoxy)NeuAcα-3Galβ-4Glcβ-Cer36 | - | - |
| 56 | GSC-77 | (8-deoxy)NeuAcα-3Galβ-4Glcβ-Cer36 | - | - |
| 57 | GSC-153 | (4,8-deoxy)NeuAcα-3Galβ-4Glcβ-Cer36 | - | - |
| 58 | GSC-51 | (9-deoxy)NeuAcα-3Galβ-4Glcβ-Cer36 | - | - |
| 59 | GSC-78 | (4-OMe)NeuAcα-3Galβ-4Glcβ-Cer36 | - | - |
| 60 | GSC-79 | (9-OMe)NeuAcα-3Galβ-4Glcβ-Cer36 | - | - |
| 61 | GSC-23 | (C7)NeuAcα-3Galβ-4Glcβ-Cer36 | - | - |
| 62 | GSC-24 | (C8)NeuAcα-3Galβ-4Glcβ-Cer36 | - | - |
| 63 | GSC-50 | (C8 diastereoisomer)NeuAcα-3Galβ-4Glcβ-Cer36 | - | - |
| 64 | GSC-229 | NeuAcα-8NeuAcα-3Galβ-4Glcβ-Cer36 | - | - |
| 65 | GSC-96 | NeuAcα-9NeuAcα-3Galβ-4Glcβ-Cer36 | - | - |
| 66 | GSC-437 | NeuAcα-8NeuAcα-8NeuAcα-3Galβ-4Glcβ-Cer36 | - | - |
| 67 | Neuα-(3')Lac | Neuα-3Galβ-4Glc-DH | - | - |
| 68 | Neuα-(3')Lac-AO | Neuα-3Galβ-4Glc-AO | - | - |
| 69 | NeuAcα-(6')Lac-AO | NeuAcα-6Galβ-4Glc-AO | - | - |
| 70 | GSC-61 | NeuAcα-6Galβ-4Glcβ-Cer36 | - | - |
| 71 | GSC-12 | NeuAcα-(S)-6Galβ-4Glcβ-Cer36 | - | - |
| 72 | GSC-234 | NeuAcα-(S)-6Galβ-(S)-4Glcβ-Cer36 | - | - |
| 73 | GSC-73 | NeuAcα-(S)-6Galβ-4Glcβ-(S)-Cer36 | - | - |
| 74 | Neuα-(6')Lac | Neuα-6Galβ-4Glc-DH | - | - |
| 75 | Neuα-(6')Lac-AO | Neuα-6Galβ-4Glc-AO | - | - |
| 76 | NeuAcβ-(3')Lac | NeuAcβ-3Galβ-4Glc-DH | - | - |
| 77 | NeuAcβ-(3')Lac-AO | NeuAcβ-3Galβ-4Glc-AO | - | - |
| 78 | NeuAcβ-(6')Lac | NeuAcβ-6Galβ-4Glc-DH | - | - |
| 79 | NeuAcβ-(6')Lac-AO | NeuAcβ-6Galβ-4Glc-AO | - | - |
| 80 | GSC-161 | NeuAcα-3Galβ-4Glcβ-C30  │  Fucα-3 | - | - |
| 81 | GSC-162 | NeuAcα-3Galβ-4Glcβ-Cer36  │  Fucα-3 | - | - |
| 82 | LacNAc(1-3) | Galβ-3GlcNAc-DH | - | - |
| 83 | LacNAc(1-3)-AO | Galβ-3GlcNAc-AO | - | - |
| 84 | LacNAc | Galβ-4GlcNAc-DH | - | - |
| 85 | LacNAc-AO | Galβ-4GlcNAc-AO | - | - |
| 86 | Galα-4Galβ-4GlcNAc | Galα-4Galβ-4GlcNAc-DH | - | - |
| 87 | SU(3')-LN | SU-3Galβ-4GlcNAc-DH | - | - |
| 88 | Lea-Tri | Galβ-3GlcNAc-DH  │  Fucα-4 | - | - |
| 89 | Lea-Tri-AO | Galβ-3GlcNAc-AO  │  Fucα-4 | - | - |
| 90 | Lex-Tri | Galβ-4GlcNAc-DH  │  Fucα-3 | - | - |
| 91 | Lex-Tri-AO | Galβ-4GlcNAc-AO  │  Fucα-3 | - | - |
| 92 | Lex-Tri-(Me)AO | Galβ-4GlcNAc-(Me)AO  │  Fucα-3 | - | - |
| 93 | SU(3')-Lea-Tri | SU-3Galβ-3GlcNAc-DH  │  Fucα-4 | - | - |
| 94 | SU(3')-Lex-Tri | SU-3Galβ-4GlcNAc-DH  │  Fucα-3 | - | - |
| 95 | NeuAcα-(3')LN | NeuAcα-3Galβ-4GlcNAc-DH | - | - |
| 96 | NeuAcα-(3')LN-AO | NeuAcα-3Galβ-4GlcNAc-AO | - | - |
| 97 | PI-1 | NeuAcα-3(6-NAc)Galβ-4GlcNAc-DH | - | - |
| 98 | PI-1-AO | NeuAcα-3(6-NAc)Galβ-4GlcNAc-AO | - | - |
| 99 | PI-2 | NeuAcα-3(6-NBz)Galβ-4GlcNAc-DH | - | - |
| 100 | PI-2-AO | NeuAcα-3(6-NBz)Galβ-4GlcNAc-AO | - | - |
| 101 | NeuAcα-(6')LN | NeuAcα-6Galβ-4GlcNAc-DH | - | - |
| 102 | NeuAcα-(6')LN-AO | NeuAcα-6Galβ-4GlcNAc-AO | - | - |
| 103 | Neu5,9Ac-(6')LN | (9-OAc)NeuAcα-6Galβ-4GlcNAc-DH | - | - |
| 104 | SA(3')-Lea-Tri | NeuAcα-3Galβ-3GlcNAc-DH  │  Fucα-4 | - | - |
| 105 | SA(3')-Lea-Tri-AO | NeuAcα-3Galβ-3GlcNAc-AO  │  Fucα-4 | - | - |
| 106 | SA(3')-Lex-Tri | NeuAcα-3Galβ-4GlcNAc-DH  │  Fucα-3 | - | - |
| 107 | SA(3')-Lex-Tri-AO | NeuAcα-3Galβ-4GlcNAc-AO  │  Fucα-3 | - | - |
| 108 | GSC-440 | NeuAcα-3Galβ-4GlcNAcβ-C30  │  Fucα-3 | - | - |
| 109 | GSC-512 | (4-OAc)NeuAcα-3Galβ-4GlcNAcβ-C30  │  Fucα-3 | - | - |
| 110 | GSC-513 | (9-OAc)NeuAcα-3Galβ-3GlcNAcβ-C30  │  Fucα-4 | - | - |
| 111 | GSC-511 | (9-OAc)NeuAcα-3Galβ-4GlcNAcβ-C30  │  Fucα-3 | - | - |
| ***Lacto-N-neotetraose and Lacto-N-tetraose-based*** | | | | |
| 112 | GSC-225 | (3-deoxy,3-carboxymethyl)Galβ-4GlcNAcβ-3Galβ-Cer36  │  Fucα-3 | - | - |
| 113 | GSC-236 | SU-3Galβ-4GlcNAcβ-3Galβ-C30  │  Fucα-3 | - | - |
| 114 | GSC-479 | NeuAcα-3Galβ-4GlcNAcβ-3Galβ-C30  │  Fucα-3 | - | - |
| 115 | GSC-105 | NeuAcα-3Galβ-4GlcNAcβ-3Galβ-Cer36  │  Fucα-3 | - | - |
| 116 | GSC-121 | NeuAcα-3Galβ-4GlcNAcβ-3Galβ-Cer36  │ (3-deoxy)Fucα-3 | - | - |
| 117 | GSC-123 | NeuAcα-3Galβ-4GlcNAcβ-3Galβ-Cer36  │ (4-deoxy)Fucα-3 | - | - |
| 118 | GSC-133 | NeuAcα-3Galβ-4GlcNAcβ-3Galβ-Cer36  │  (2-OMe)Fucα-3 | - | - |
| 119 | GSC-131 | NeuAcα-3Galβ-4GlcNAcβ-3Galβ-Cer36  │  Quvα-3 | - | - |
| 120 | GSC-163 | NeuAcα-3Galβ-4GlcNAcβ-3Galβ-Cer36   │  Rhaα-3 | - | - |
| 121 | GSC-127 | NeuAcα-3Galβ-4GlcNAcβ-3Galβ-Cer36  │ (6-deoxy)Talα-3 | - | - |
| 122 | GSC-341 | KDNα-3Galβ-4GlcNAcβ-3Galβ-C30  │   Fucα-3 | - | - |
| 123 | GSC-177 | NeuGcα-3Galβ-4GlcNAcβ-3Galβ-Cer36   │   Fucα-3 | - | - |
| 124 | GSC-175 | NeuAcα-3(4-deoxy)Galβ-4GlcNAcβ-3Galβ-Cer36  │  Fucα-3 | - | - |
| 125 | GSC-176 | NeuAcα-3(6-deoxy)Galβ-4GlcNAcβ-3Galβ-Cer36  │  Fucα-3 | - | - |
| 126 | GSC-257 | NeuAcα-3(4,6-deoxy)Galβ-4GlcNAcβ-3Galβ-Cer36  │  Fucα-3 | - | - |
| 127 | DLNN | GlcNAcβ-3Galβ-4Glc-DH | - | - |
| 128 | LNT | Galβ-3GlcNAcβ-3Galβ-4Glc-DH | - | - |
| 129 | Paragloboside | Galβ-4GlcNAcβ-3Galβ-4Glcβ-Cer | - | - |
| 130 | LNnT | Galβ-4GlcNAcβ-3Galβ-4Glc-DH | - | - |
| 131 | B-like pentaosylceramide | Galα-3Galβ-4GlcNAcβ-3Galβ-4Glcβ-Cer | - | - |
| 132 | Klaus glycolipid | Galβ-3Galβ-4GlcNAcβ-3Galβ-4Glcβ-Cer | - | - |
| 133 | GSC-207 | GlcAβ-3Galβ-4GlcNAcβ-3Galβ-4Glcβ-C30 | - | - |
| 134 | GSC-191 | GlcAβ-3Galβ-4GlcNAcβ-3Galβ-4Glcβ-Cer36 | - | - |
| 135 | GSC-189 | GlcAβ-3Galβ-4GlcNAcβ-3Galβ-4Glcβ-Cer42 | - | - |
| 136 | SU(3')-Tri | SU-3Galβ-4GlcNAcβ-3Gal-DH | - | - |
| 137 | GSC-208 | SU-3GlcAβ-3Galβ-4GlcNAcβ-3Galβ-4Glcβ-C30 | - | - |
| 138 | GSC-192 | SU-3GlcAβ-3Galβ-4GlcNAcβ-3Galβ-4Glcβ-Cer36 | - | - |
| 139 | GSC-190 | SU-3GlcAβ-3Galβ-4GlcNAcβ-3Galβ-4Glcβ-Cer42 | - | - |
| 140 | Led-II pentaosylceramide | Fucα-2Galβ-3GlcNAcβ-3Galβ-4Glcβ-CerA | - | - |
| 141 | Led-I pentaosylceramide | Fucα-2Galβ-3GlcNAcβ-3Galβ-4Glcβ-CerB | - | - |
| 142 | LNFP-I | Fucα-2Galβ-3GlcNAcβ-3Galβ-4Glc-DH | - | - |
| 143 | B-hexaosylceramide | Galα-3Galβ-4GlcNAcβ-3Galβ-4Glcβ-Cer  │  Fucα-2 | - | - |
| 144 | A-Hexa | GalNAcα-3Galβ-3GlcNAcβ-3Galβ-4Glc-DH  │  Fucα-2 | - | - |
| 145 | A-Hepta | Fucα-4  │ GalNAcα-3Galβ-3GlcNAcβ-3Galβ-4Glc-DH  │  Fucα-2 | - | - |
| 146 | LNFP-II | Galβ-3GlcNAcβ-3Galβ-4Glc-DH  │  Fucα-4 | - | - |
| 147 | LNDFH-II | Galβ-3GlcNAcβ-3Galβ-4Glc-DH  │ │  Fucα-4 Fucα-3 | - | - |
| 148 | Leb-hexaosylceramide | Fucα-2Galβ-3GlcNAcβ-3Galβ-4Glcβ-Cer  │  Fucα-4 | - | - |
| 149 | LNDFH-I | Fucα-2Galβ-3GlcNAcβ-3Galβ-4Glc-DH  │  Fucα-4 | - | - |
| 150 | LNTFH-I | Fucα-2Galβ-3GlcNAcβ-3Galβ-4Glc-DH  │ │  Fucα-4 Fucα-2 | - | - |
| 151 | LNFP-III | Galβ-4GlcNAcβ-3Galβ-4Glc-DH  │  Fucα-3 | - | - |
| 152 | LNFP-III-AO | Galβ-4GlcNAcβ-3Galβ-4Glc-AO  │  Fucα-3 | - | - |
| 153 | LNnDFH-I | Fucα-2Galβ-4GlcNAcβ-3Galβ-4Glc-DH  │  Fucα-3 | - | - |
| 154 | LNnDFH-II | Galβ-4GlcNAcβ-3Galβ-4Glc-DH  │ │  Fucα-3 Fucα-3 | - | - |
| 155 | LNnDFH-V | Galβ-4GlcNAcβ-3Galβ-4Glc-DH  │ │  Fucα-3 Fucα-2 | - | - |
| 156 | LNnTFH-I | Fucα-2Galβ-4GlcNAcβ-3Galβ-4Glc-DH  │ │  Fucα-3 Fucα-2 | - | - |
| 157 | SU(3')-LNFP-II | SU-3Galβ-3GlcNAcβ-4Galβ-4Glc-DH  │  Fucα-4 | - | - |
| 158 | SU(6')-LNFP-II | SU-6Galβ-3GlcNAcβ-3Galβ-4Glc-DH  │  Fucα-4 | - | - |
| 159 | SU(3')-LNFP-III | SU-3Galβ-4GlcNAcβ-3Galβ-4Glc-DH  │  Fucα-3 | - | - |
| 160 | SU(6')-LNFP-III | SU-6Galβ-4GlcNAcβ-3Galβ-4Glc-DH  │  Fucα-3 | - | - |
| 161 | SU(3',6)-LNFP-III | SU-6  │ SU-3Galβ-4GlcNAcβ-3Galβ-4Glc-DH  │  Fucα-3 | - | - |
| 162 | LSTa | NeuAcα-3Galβ-3GlcNAcβ-3Galβ-4Glc-DH | - | - |
| 163 | GSC-272 | NeuAcα-3Galβ-3GlcNAcβ-3Galβ-4Glcβ-C30 | - | - |
| 164 | GSC-147 | KDNα-3Galβ-3GlcNAcβ-3Galβ-4Glcβ-Cer36 | - | - |
| 165 | GSC-396 | NeuGcα-3Galβ-3GlcNAcβ-3Galβ-4Glcβ-C30 | - | - |
| 166 | LSTb | Galβ-3GlcNAcβ-3Galβ-4Glc-DH  │ NeuAcα-6 | - | - |
| 167 | GSC-397 | NeuGcα-6Galβ-3GlcNAcβ-3Galβ-4Glcβ-C30 | - | - |
| 168 | DSLNT | NeuAcα-3Galβ-3GlcNAcβ-3Galβ-4Glc-DH  │  NeuAcα-6 | - | - |
| 169 | Sialylparagloboside | NeuAcα-3Galβ-4GlcNAcβ-3Galβ-4Glcβ-Cer | - | - |
| 170 | GSC-273 | NeuAcα-3Galβ-4GlcNAcβ-3Galβ-4Glcβ-C30 | - | - |
| 171 | GSC-31 | NeuAcα-3Galβ-4GlcNAcβ-3Galβ-4Glcβ-Cer36 | - | - |
| 172 | LSTc | NeuAcα-6Galβ-4GlcNAcβ-3Galβ-4Glc-DH | - | - |
| 173 | GSC-516B | Neuα-3Galβ-4GlcNAcβ-3Galβ-4Glcβ-Cer36  │  SU-6 | - | - |
| 174 | SA(3/6)LNFP-I | NeuAcα-3/6Galβ-3GlcNAcβ-3Galβ-4Glc-DH  │  Fucα-2 | - | - |
| 175 | SA(3')-LNFP-II | NeuAcα-3Galβ-3GlcNAcβ-3Galβ-4Glc-DH  │  Fucα-4 | - | - |
| 176 | SA(6')-LNFP-VI | NeuAcα-6Galβ-4GlcNAcβ-3Galβ-4Glc-DH  │  Fucα-3 | - | - |
| 177 | GSC-533 | NeuAcα-3Galβ-4GlcNβ-3Galβ-4Glcβ-Cer36  │  Fucα-3 | - | - |
| 178 | GSC-64 | NeuAcα-3Galβ-4GlcNAcβ-3Galβ-4Glcβ-Cer36  │  Fucα-3 | - | - |
| 179 | SA(3')-LNFP-III | NeuAcα-3Galβ-4GlcNAcβ-3Galβ-4Glc-DH  │  Fucα-3 | - | - |
| 180 | GSC-472 | Neuα-3Galβ-4GlcNAcβ-3Galβ-4Glcβ-Cer36  │  Fucα-3 | - | - |
| 181 | GSC-97 | NeuAcα-6Galβ-4GlcNAcβ-3Galβ-4Glcβ-Cer36  │  Fucα-3 | - | - |
| 182 | GSC-314 | KDNα-3Galβ-4GlcNAcβ-3Galβ-4Glcβ-C30  │  Fucα-3 | - | - |
| 183 | GSC-149 | KDNα-3Galβ-4GlcNAcβ-3Galβ-4Glcβ-Cer36  │   Fucα-3 | - | - |
| 184 | GSC-311 | KDNα-3Galβ-4GlcNAcβ-3Galβ-4Glcβ-C30  │  Rhaα-3 | - | - |
| 185 | GSC-268 | SU-6  │ NeuAcα-3Galβ-4GlcNAcβ-3Galβ-4Glcβ-Cer36  │  Fucα-3 | - | - |
| 186 | GSC-268 deNAc | SU-6  │ Neuα-3Galβ-4GlcNβ-3Galβ-4Glcβ-Cer36  │  Fucα-3 | - | - |
| 187 | GSC-269 | SU-6  │ NeuAcα-3Galβ-4GlcNAcβ-3Galβ-4Glcβ-Cer36  │  Fucα-3 | - | - |
| 188 | GSC-406 | SU-6  │ Neuα-3Galβ-4GlcNAcβ-3Galβ-4Glcβ-Cer36  │  Fucα-3 | - | - |
| 189 | GSC-270 | SU-6 SU-6  │ │ NeuAcα-3Galβ-4GlcNAcβ-3Galβ-4Glcβ-Cer36  │  Fucα-3 | - | - |
| ***Polylactosamine Ii-active*** | | | | |
| 190 | pLNH | Galβ-3GlcNAcβ-3Galβ-4GlcNAcβ-3Galβ-4Glc-DH | - | - |
| 191 | pLNnH | Galβ-4GlcNAcβ-3Galβ-4GlcNAcβ-3Galβ-4Glc-DH | - | - |
| 192 | GSC-216 | GlcAβ-3Galβ-4GlcNAcβ-3Galβ-4GlcNAcβ-3Galβ-4Glcβ-Cer42 | - | - |
| 193 | GSC-217 | SU-3GlcAβ-3Galβ-4GlcNAcβ-3Galβ-4GlcNAcβ-3Galβ-4Glcβ-Cer42 | - | - |
| 194 | GSC-218 | GlcAβ-3Galβ-4GlcNAcβ-3Galβ-4GlcNAcβ-3Galβ-4Glcβ-Cer36 | - | - |
| 195 | GSC-219 | SU-3GlcAβ-3Galβ-4GlcNAcβ-3Galβ-4GlcNAcβ-3Galβ-4Glcβ-Cer36 | - | - |
| 196 | LNH | Galβ-4GlcNAcβ-6  │  Galβ-4Glc-DH  │ Galβ-3GlcNAcβ-3 | - | - |
| 197 | iLNO | Galβ-3GlcNAcβ-3Galβ-4GlcNAcβ-6  │  Galβ-4Glc-DH  │  Galβ-3GlcNAcβ-3 | - | - |
| 198 | LND | Galβ-4GlcNAcβ-6  │  Galβ-4GlcNAcβ-6  │ │ Galβ-3GlcNAcβ-3 Galβ-4Glc-DH  │  Galβ-3GlcNAcβ-3 | - | - |
| 199 | LNnH | Galβ-4GlcNAcβ-6  │  Galβ-4Glc-DH  │ Galβ-4GlcNAcβ-3 | - | - |
| 200 | Nonaosylceramide | GlcNAcβ-6  │ GlcNAcβ-6 Galβ-4GlcNAcβ-3Galβ-4Glcβ-Cer  │ │  Galβ-4GlcNAcβ-3  │ GlcNAcβ-3 | - | - |
| 201 | I-octaosylceramide | Galβ-4GlcNAcβ-6  │  Galβ-4GlcNAcβ-3Galβ-4Glcβ-Cer  │ Galβ-4GlcNAcβ-3 | - | - |
| 202 | I-dodecaosylceramide | Galβ-4GlcNAcβ-6  │ Galβ-4GlcNAcβ-6 Galβ-4GlcNAcβ-3Galβ-4Glcβ-Cer  │ │  Galβ-4GlcNAcβ-3  │ Galβ-4GlcNAcβ-3 | - | - |
| 203 | I-hexadecaosylceramide | Galβ-4GlcNAcβ-6  │  Galβ-4GlcNAcβ-6 Galβ-4GlcNAcβ-3Galβ-4Glcβ-Cer  │ │ Galβ-4GlcNAcβ-6 Galβ-4GlcNAcβ-3  │ │  Galβ-4GlcNAcβ-3  │ Galβ-4GlcNAcβ-3 | - | - |
| 204 | I-eicosaosylceramide | Galβ-4GlcNAcβ-6  │  Galβ-4GlcNAcβ-6 Galβ-4GlcNAcβ-3Galβ-4Glcβ-Cer  │ │  Galβ-4GlcNAcβ-6 Galβ-4GlcNAcβ-3  │ │ Galβ-4GlcNAcβ-6 Galβ-4GlcNAcβ-3  │ │  Galβ-4GlcNAcβ-3  │ Galβ-4GlcNAcβ-3 | - | - |
| 205 | B-like decaosylceramide | Galα-3Galβ-4GlcNAcβ-6  │  Galβ-4GlcNAcβ-3Galβ-4Glcβ-Cer  │ Galα-3Galβ-4GlcNAcβ-3 | - | - |
| 206 | B-like pentadecaosylceramide | Galα-3Galβ-4GlcNAcβ-6  │ Galα-3Galβ-4GlcNAcβ-6 Galβ-4GlcNAcβ-3Galβ-4Glcβ-Cer  │ │  Galβ-4GlcNAcβ-3  │ Galα-3Galβ-4GlcNAcβ-3 | - | - |
| 207 | B-like eicosaosylceramide | Galα-3Galβ-4GlcNAcβ-6  │  Galα-3Galβ-4GlcNAcβ-6 Galβ-4GlcNAcβ-3Galβ-4Glcβ-Cer  │ │ Galα-3Galβ-4GlcNAcβ-6 Galβ-4GlcNAcβ-3  │ │  Galβ-4GlcNAcβ-3  │ Galα-3Galβ-4GlcNAcβ-3 | - | - |
| 208 | B-like pentaeicosaosylceramide | Galα-3Galβ-4GlcNAcβ-6  │  Galα-3Galβ-4GlcNAcβ-6 Galβ-4GlcNAcβ-3Galβ-4Glcβ-Cer  │ │  Galα-3Galβ-4GlcNAcβ-6 Galβ-4GlcNAcβ-3  │ │ Galα-3Galβ-4GlcNAcβ-6 Galβ-4GlcNAcβ-3  │ │  Galβ-4GlcNAcβ-3  │ Galα-3Galβ-4GlcNAcβ-3 | - | - |
| 209 | pLNFH-IV | Galβ-3GlcNAcβ-3Galβ-4GlcNAcβ-3Galβ-4Glc-DH  │  Fucα-3 | - | - |
| 210 | DFpLNH-II | Galβ-3GlcNAcβ-3Galβ-4GlcNAcβ-3Galβ-4Glc-DH  │ │  Fucα-4 Fucα-3 | - | - |
| 211 | TFpLNH-I | Fucα-2Galβ-3GlcNAcβ-3Galβ-4GlcNAcβ-3Galβ-4Glc-DH  │ │  Fucα-4 Fucα-3 | - | - |
| 212 | MFLNH-III | Galβ-4GlcNAcβ-6  │ │  Fucα-3 Galβ-4Glc-DH  │ Galβ-3GlcNAcβ-3 | - | - |
| 213 | DFLNH(b) | Galβ-4GlcNAcβ-6  │ │  Fucα-3 Galβ-4Glc-DH  │ Galβ-3GlcNAcβ-3  │  Fucα-4 | - | - |
| 214 | DFLNH(c) | Galβ-4GlcNAcβ-6  │  Galβ-4Glc-DH  │ Fucα-2Galβ-3GlcNAcβ-3  │  Fucα-4 | - | - |
| 215 | DFLNH(a) | Galβ-4GlcNAcβ-6  │ │  Fucα-3 Galβ-4Glc-DH  │ Fucα-2Galβ-3GlcNAcβ-3 | - | - |
| 216 | TFLNH | Galβ-4GlcNAcβ-6   │ │   Fucα-3 Galβ-4Glc-DH  │  Fucα-2Galβ-3GlcNAcβ-3   │   Fucα-4 | - | - |
| 217 | MFiLNO-IV | Galβ-3GlcNAcβ-3Galβ-4GlcNAcβ-6  │ │  Fucα-3 Galβ-4Glc-DH  │  Galβ-3GlcNAcβ-3 | - | - |
| 218 | TFiLNO | Galβ-3GlcNAcβ-3Galβ-4GlcNAcβ-6  │ │ │  Fucα-4 Fucα-3 Galβ-4Glc-DH  │  Galβ-3GlcNAcβ-3  │  Fucα-4 | - | - |
| 219 | MFLND | Galβ-4GlcNAcβ-6  │ │  Fucα-3 Galβ-4GlcNAcβ-6  │ │ Galβ-3GlcNAcβ-3 Galβ-4Glc-DH  │  Galβ-3GlcNAcβ-3 | - | - |
| 220 | MFLNnH(a) | Galβ-4GlcNAcβ-6  │ │  Fucα-3 Galβ-4Glc-DH  │ Galβ-4GlcNAcβ-3 | - | - |
| 221 | DFLNnH | Galβ-4GlcNAcβ-6  │ │  Fucα-3 Galβ-4Glc-DH  │ Galβ-4GlcNAcβ-3  │  Fucα-3 | - | - |
| 222 | B-III dodecaosylceramide | Galα-3Galβ-4GlcNAcβ-6  │ │  Fucα-2 Galβ-4GlcNAcβ-3Galβ-4Glcβ-Cer  │ Galα-3Galβ-4GlcNAcβ-3  │  Fucα-2 | - | - |
| 223 | B-IV tetradecaosylceramide | Galα-3Galβ-4GlcNAcβ-6  │ │  Fucα-2 Galβ-4GlcNAcβ-3Galβ-4Glcβ-Cer  │ Galα-3Galβ-4GlcNAcβ-3Galβ-4GlcNAcβ-3  │  Fucα-2 | - | - |
| 224 | MSLNH | NeuAcα-6Galβ-4GlcNAcβ-6  │  Galβ-4Glc-DH  │  Galβ-3GlcNAcβ-3 | - | - |
| 225 | MSLNnH-I | Galβ-4GlcNAcβ-6  │  Galβ-4Glc-DH  │ NeuAcα-6Galβ-3GlcNAcβ-3 | - | - |
| 226 | DSLNnH | NeuAcα-6Galβ-4GlcNAcβ-6  │  Galβ-4Glc-DH  │ NeuAcα-6Galβ-4GlcNAcβ-3 | - | - |
| 227 | MSMFLNH | Galβ-4GlcNAcβ-6  │ │  Fucα-3 Galβ-4Glc-DH  │ NeuAcα-3Galβ-3GlcNAcβ-3 | - | - |
| 228 | MFMSLNnH | Galβ-4GlcNAcβ-6  │ │  Fucα-3 Galβ-4Glc-DH  │ NeuAcα-6Galβ-3GlcNAcβ-3 | - | - |
| 229 | GSC-221 | NeuAcα-3Galβ-4GlcNAcβ-3Galβ-4GlcNAcβ-3Galβ-4Glcβ-Cer36  │  Fucα-3 | - | - |
| 230 | GSC-220 | NeuAcα-3Galβ-4GlcNAcβ-3Galβ-4GlcNAcβ-3Galβ-4Glcβ-Cer36  │ │  Fucα-3 Fucα-3 | - | - |
| 231 | C4U | NeuAcα-3Galβ-4GlcNAcβ-3Galβ-3GlcNAc-DH  │ │ │  SU-6 SU-6 SU-6 | - | - |
| ***N-glycans*** | | | | |
| 232 | Man2(α2) | Manα-2Man-DH | - | - |
| 233 | Man2(α3) | Manα-3Man-DH | - | - |
| 234 | Man2(α6) | Manα-6Man-DH | - | - |
| 235 | Man3(α3,α6) | Manα-6Man-DH  │  Manα-3 | - | - |
| 236 | Man5(α3,α6) | Manα-3  │ Manα-6Manα-6Man-DH  │  Manα-3 | - | - |
| 237 | Man1GN1 | Manβ-4GlcNAc-DH | - | - |
| 238 | Man2GN1 | Manα-3Manβ-4GlcNAc-DH | - | - |
| 239 | Man2aGN2 | Manα-6Manβ-4GlcNAcβ-4GlcNAc-DH | - | - |
| 240 | Man3GN2 | Manα-6  │  Manβ-4GlcNAcβ-4GlcNAc-DH  │ Manα-3 | - | - |
| 241 | Man4aGN2 | Manα-3Manα-6  │  Manβ-4GlcNAcβ-4GlcNAc-DH  │  Manα-3 | - | - |
| 242 | Man4bGN2 | Manα-6  │ Manα-3Manα-6  │  Manβ-4GlcNAcβ-4GlcNAc-DH | - | - |
| 243 | Man5GN2 | Manα-6  │ Manα-3Manα-6  │  Manβ-4GlcNAcβ-4GlcNAc-DH  │  Manα-3 | - | - |
| 244 | Man6GN2 | Manα-6  │ Manα-3Manα-6  │  Manβ-4GlcNAcβ-4GlcNAc-DH  │ Manα-2Manα-3 | - | - |
| 245 | Man7(D1)GN2 | Manα-6  │  Manα-3Manα-6  │  Manβ-4GlcNAcβ-4GlcNAc-DH  │ Manα-2Manα-2Manα-3 | - | - |
| 246 | Man7(D1)GN2-AO | Manα-6  │  Manα-3Manα-6  │  Manβ-4GlcNAcβ-4GlcNAc-AO  │ Manα-2Manα-2Manα-3 | - | - |
| 247 | Man7(D3)GN2 | Manα-2Manα-6  │  Manα-3Manα-6  │  Manβ-4GlcNAcβ-4GlcNAc-DH  │  Manα-2Manα-3 | - | - |
| 248 | Man8(D1D3)GN2 | Manα-2Manα-6  │  Manα-3Manα-6  │  Manβ-4GlcNAcβ-4GlcNAc-DH  │ Manα-2Manα-2Manα-3 | - | - |
| 249 | Man9GN2 | Manα-2Manα-6  │ Manα-2Manα-3Manα-6  │  Manβ-4GlcNAcβ-4GlcNAc-DH  │ Manα-2Manα-2Manα-3 | - | - |
| 250 | Man9GN2-AO | Manα-2Manα-6  │ Manα-2Manα-3Manα-6  │  Manβ-4GlcNAcβ-4GlcNAc-AO  │ Manα-2Manα-2Manα-3 | - | - |
| 251 | Glc1Man9GN2 | Manα-2Manα-6  │  Manα-6  │ │  Manα-2Manα-3 Manβ-4GlcNAcβ-4GlcNAc-DH  │ Glcα-3Manα-2Manα-2Manα-3 | - | - |
| 252 | Glc1Man9GN2-AO | Manα-2Manα-6  │  Manα-6  │ │  Manα-2Manα-3 Manβ-4GlcNAcβ-4GlcNAc-AO  │ Glcα-3Manα-2Manα-2Manα-3 | - | - |
| 253 | Glc2Man7(D1)GN1-AO | Manα-6  │  Manα-3Manα-6  │  Manβ-4GlcNAc-AO  │ Glcα-3Glcα-3Manα-2Manα-2Manα-3 | - | - |
| 254 | Glc3Man7(D1)GN1-AO | Manα-6  │  Manα-3Manα-6  │  Manβ-4GlcNAc-AO  │ Glcα-2Glcα-3Glcα-3Manα-2Manα-2Manα-3 | - | - |
| 255 | Man3XylGN2 | Manα-6  │ Xylβ-2Manβ-4GlcNAcβ-4GlcNAc-DH  │  Manα-3 | - | - |
| 256 | Man3XylGN2 | Manα-6  │ Xylβ-2Manβ-4GlcNAcβ-4GlcNAc-DH  │  Manα-3 | - | - |
| 257 | N1 | Galβ-4GlcNAcβ-2Manα-6 Fucα-6  │ │  Manβ-4GlcNAcβ-4GlcNAc-DH  │  Manα-3 | - | - |
| 258 | N2 | Manα-6  │  Manβ-4GlcNAcβ-4GlcNAc-DH  │ Galβ-4GlcNAcβ-2Manα-3 | - | - |
| 259 | N4 | Galβ-4GlcNAcβ-2Manα-6  │  Manβ-4GlcNAcβ-4GlcNAc-DH  │  Manα-3 | - | - |
| 260 | GlcNac2Man3-AO | GlcNAcβ-2Manα-6  │  Man-AO  │ GlcNAcβ-2Manα-3 | - | - |
| 261 | N3 | GlcNAcβ-2Manα-6 Fucα-6  │ │ Galβ-4 Manβ-4GlcNAcβ-4GlcNAc-DH  │  GlcNAcβ-2Manα-3 | - | - |
| 262 | NGA2 | GlcNAcβ-2Manα-6  │  Manβ-4GlcNAcβ-4GlcNAc-DH  │ GlcNAcβ-2Manα-3 | - | - |
| 263 | NGA2B | GlcNAcβ-2Manα-6  │  GlcNAcβ-4Manβ-4GlcNAcβ-4GlcNAc-DH  │ GlcNAcβ-2Manα-3 | - | - |
| 264 | NGA3B | GlcNAcβ-2Manα-6  │  GlcNAcβ-4Manβ-4GlcNAcβ-4GlcNAc-DH  │ GlcNAcβ-4Manα-3  │  GlcNAcβ-2 | - | - |
| 265 | NGA4 | GlcNAcβ-6  │ GlcNAcβ-2Manα-6  │  Manβ-4GlcNAcβ-4GlcNAc-DH  │ GlcNAcβ-2Manα-3  │  GlcNAcβ-4 | - | - |
| 266 | NGA5B | GlcNAcβ-2  │ GlcNAcβ-4Manα-6  │ │  GlcNAcβ-6 │  │  GlcNAcβ-4Manβ-4GlcNAcβ-4GlcNAc-DH  │ GlcNAcβ-4Manα-3  │  GlcNAcβ-2 | - | - |
| 267 | GNMan5BGN2 | Manα-6  │  Manα-3Manα-6  │  GlcNAcβ-4Manβ-4GlcNAcβ-4GlcNAc-DH  │ GlcNAcβ-2Manα-3 | - | - |
| 268 | NA2 | Galβ-4GlcNAcβ-2Manα-6  │  Manβ-4GlcNAcβ-4GlcNAc-DH  │ Galβ-4GlcNAcβ-2Manα-3 | - | - |
| 269 | NA3 | Galβ-4GlcNAcβ-2Manα-6   │   Manβ-4GlcNAcβ-4GlcNAc-DH  │  Galβ-4GlcNAcβ-4Manα-3   │   Galβ-4GlcNAcβ-2 | - | - |
| 270 | NA4 | Galβ-4GlcNAcβ-6  │ Galβ-4GlcNAcβ-2Manα-6  │  Manβ-4GlcNAcβ-4GlcNAc-DH  │ Galβ-4GlcNAcβ-4Manα-3  │   Galβ-4GlcNAcβ-2 | - | - |
| 271 | Fuc-GlcNAc | Fucα-6GlcNAc-DH | - | - |
| 272 | Man3FGN2 | Manα-6 Fucα-6  │ │  Manβ-4GlcNAcβ-4GlcNAc-DH  │ Manα-3 | - | - |
| 273 | Man3FXylGN2 | Manα-6  │ Xylβ-2Manα-4GlcNAcβ-4GlcNAc-DH  │ │  Manα-3 Fucα-3 | - | - |
| 274 | NGA2F | GlcNAcβ-2Manα-6 Fucα-6  │ │  Manβ-4GlcNAcβ-4GlcNAc-DH  │ GlcNAcβ-2Manα-3 | - | - |
| 275 | NA2F-AO | Galβ-4GlcNAcβ-2Manα-6 Fucα-6  │ │  Manβ-4GlcNAcβ-4GlcNAc-AO  │ Galβ-4GlcNAcβ-2Manα-3 | - | - |
| 276 | NA2FB | Galβ-4GlcNAcβ-2Manα-6 Fucα-6  │ │  GlcNAcβ-4Manβ-4GlcNAcβ-4GlcNAc-DH  │ Galβ-4GlcNAcβ-2Manα-3 | - | - |
| 277 | NA3-Lex | Galβ-4GlcNAcβ-2Manα-6  │ Fucα-3 Manβ-4GlcNAcβ-4GlcNAc-DH  │  Galβ-4GlcNAcβ-4Manα-3  │  Galβ-4GlcNAcβ-2 | - | - |
| 278 | A2(2-6) | NeuAcα-6Galβ-4GlcNAcβ-2Manα-6  │  Manβ-4GlcNAcβ-4GlcNAc-DH  │ NeuAcα-6Galβ-4GlcNAcβ-2Manα-3 | - | - |
| 279 | AGP-Bi-Ac2 | NeuAcα-6Galβ-4GlcNAcβ-2Manα-6  │  Manβ-4GlcNAcβ-4GlcNAc-DH  │ NeuAcα-6Galβ-4GlcNAcβ-2Manα-3 | - | - |
| 280 | AGP-Bi-Gc2 | NeuGcα-6Galβ-4GlcNAcβ-2Manα-6  │  Manβ-4GlcNAcβ-4GlcNAc-DH  │ NeuGcα-6Galβ-4GlcNAcβ-2Manα-3 | - | - |
| 281 | AGP-Bi-AcGc | NeuGc(Ac)α-6Galβ-4GlcNAcβ-2Manα-6  │  Manβ-4GlcNAcβ-4GlcNAc-DH  │ NeuAc(Gc)α-6Galβ-4GlcNAcβ-2Manα-3 | - | - |
| 282 | A3 | NeuAcα-3Galβ-4GlcNAcβ-2Manα-6  │  Manβ-4GlcNAcβ-4GlcNAc-DH  │ NeuAcα-3Galβ-4GlcNAcβ-4Manα-3  │  NeuAcα-6Galβ-4GlcNAcβ-2 | - | - |
| 283 | A2F(2-3) | NeuAcα-3Galβ-4GlcNAcβ-2Manα-6 Fucα-6  │ │  Manβ-4GlcNAcβ-4GlcNAc-DH  │ NeuAcα-3Galβ-4GlcNAcβ-2Manα-3 | - | - |
| ***Ganglioside*** | | | | |
| 284 | GM4 | NeuAcα-3Galβ-Cer | - | - |
| 285 | SM3 | SU-3Galβ-4Glcβ-Cer | - | - |
| 286 | Haematoside | NeuAcα-3Galβ-4Glcβ-Cer | - | - |
| 287 | GM3 | NeuAcα-3Galβ-4Glcβ-Cer | - | - |
| 288 | GM3(Gc) | NeuGcα-3Galβ-4Glcβ-Cer | - | - |
| 289 | Asialo-GM2 | GalNAcβ-4Galβ-4Glcβ-Cer | - | - |
| 290 | SB2 | SU-3GalNAcβ-4Galβ-4Glcβ-Cer  │  SU-3 | - | - |
| 291 | GM2 | GalNAcβ-4Galβ-4Glcβ-Cer  │  NeuAcα-3 | - | - |
| 292 | GSC-576 | GalNAcβ-4Galβ-3Glcβ-C30  │  NeuAcα-3 | - | - |
| 293 | GSC-108 | GalNAcβ-4Galβ-4Glcβ-Cer36  │  NeuAcα-3 | - | - |
| 294 | GSC-193 | GalNAcβ-4Galβ-4Glcβ-Cer36   │   KDNα-3 | - | - |
| 295 | Asialo-GM1 | Galβ-3GalNAcβ-4Galβ-4Glcβ-Cer | - | - |
| 296 | Asialo-GM1-Tetra | Galβ-3GalNAcβ-4Galβ-4Glc-DH | - | - |
| 297 | SM1a | Galβ-3GalNAcβ-4Galβ-4Glcβ-Cer  │  SU-3 | - | - |
| 298 | SB1a | SU-3Galβ-3GalNAcβ-4Galβ-4Glcβ-Cer  │  SU-3 | - | - |
| 299 | GSC-335 | SU-6  │ NeuAcα-3Galβ-3GalNAcβ-4Galβ-4Glcβ-Cer36 | - | - |
| 300 | GM1 | Galβ-3GalNAcβ-4Galβ-4Glcβ-Cer  │  NeuAcα-3 | - | - |
| 301 | GM1-penta | Galβ-3GalNAcβ-4Galβ-4Glc-DH  │  NeuAcα-3 | - | - |
| 302 | GM1(Gc) | Galβ-3GalNAcβ-4Galβ-4Glcβ-Cer  │  NeuGcα-3 | - | - |
| 303 | GM1(Gc)-penta | Galβ-3GalNAcβ-4Galβ-4Glc-DH  │  NeuGcα-3 | - | - |
| 304 | GD1a | NeuAcα-3Galβ-3GalNAcβ-4Galβ-4Glcβ-Cer  │  NeuAcα-3 | - | - |
| 305 | GD1a-hexa | NeuAcα-3Galβ-3GalNAcβ-4Galβ-4Glc-DH  │  NeuAcα-3 | - | - |
| 306 | GalNAc-GD1a(Ac,Gc) | GalNAcβ-4Galβ-3GalNAcβ-4Galβ-4Glcβ-Cer   │ │  NeuGcα-3 NeuAcα-3  GalNAcβ-4Galβ-3GalNAcβ-4Galβ-4Glcβ-Cer   │ │  NeuAcα-3 NeuGcα-3 | - | - |
| 307 | GSC-195 | KDNα-3Galβ-3GalNAcβ-4Galβ-4Glcβ-Cer36   │   KDNα-3 | - | - |
| 308 | GD3 | NeuAcα-8NeuAcα-3Galβ-4Glcβ-Cer | - | - |
| 309 | GD3-tetra | NeuAcα-8NeuAcα-3Galβ-4Glc-DH | - | - |
| 310 | GD3-tetra-AO | NeuAcα-8NeuAcα-3Galβ-4Glc-AO | - | - |
| 311 | GD2 | GalNAcβ-4Galβ-4Glcβ-Cer  │ NeuAcα-8NeuAcα-3 | - | - |
| 312 | GD1b | Galβ-3GalNAcβ-4Galβ-4Glcβ-Cer  │ NeuAcα-8NeuAcα-3 | - | - |
| 313 | GT1a | NeuAcα-8NeuAcα-3Galβ-3GalNAcβ-4Galβ-4Glcβ-Cer  │  NeuAcα-3 | - | - |
| 314 | GT1b | NeuAcα-3Galβ-3GalNAcβ-4Galβ-4Glcβ-Cer  │  NeuAcα-8NeuAcα-3 | - | - |
| 315 | GQ1b | NeuAcα-8NeuAcα-3Galβ-3GalNAcβ-4Galβ-4Glcβ-Cer  │  NeuAcα-8NeuAcα-3 | - | - |
| 316 | GSC-442 | GalNAcβ-4Galβ-4Glcβ-Cer36   │   NeuAcα-6 | - | - |
| 317 | GSC-68 | NeuAcα-6Galβ-3GalNAcβ-4Galβ-4Glcβ-Cer36 | - | - |
| 318 | GSC-107 | NeuAcα-6Galβ-3GalNAcβ-4Galβ-4Glcβ-Cer36   │   NeuAcα-6 | - | - |
| 319 | GSC-118 | NeuAcα-3Galβ-3GalNAcβ-4Galβ-4Glcβ-Cer36   │   NeuAcα-6 | - | - |
| ***O-glycans*** | | | | |
| 320 | GalNAc-Ser | GalNAcα-Ser-DH | - | - |
| 321 | GalNAc-Thr | GalNAcα-Thr-DH | - | - |
| 322 | BSM-Di-A1-AO | NeuGcα-6GalNAc-AO | - | - |
| 323 | BSM-Di-A2-AO | NeuAcα-6GalNAc-AO | 2177 | 59 |
| 324 | GalNAcα-3GalNAc | GalNAcα-3GalNAc-DH | - | - |
| 325 | Galβ-3GalNAc | Galβ-3GalNAc-DH | - | - |
| 326 | Galβ-3GalNAc-AO | Galβ-3GalNAc-AO | - | - |
| 327 | Galβ-6GalNAc | Galβ-6GalNAc-DH | - | - |
| 328 | Galβ-6GalNAc-AO | Galβ-6GalNAc-AO | - | - |
| 329 | Man-Ser | Manα-Ser-DH | - | - |
| 330 | Man-Ser-Succ | Manα-Ser-Succ-DH | - | - |
| 331 | Man-Thr | Manα-Thr-DH | - | - |
| 332 | Man-Thr-Succ | Manα-Thr-Succ-DH | - | - |
| 333 | A8/1 | GlcNAcα-4Galβ-OX | - | - |
| 334 | A8/2 | SU-6  │ Fucα-3GlcNAcβ-OY | - | - |
| 335 | A15/1 | SU-6GlcNAcβ-OY | - | - |
| 336 | A15/3 | GlcNAcα-4Galβ-3Galβ-OX  │  Fucα-2 | - | - |
| 337 | Notch-1 | Fucα-Thr-DH | - | - |
| 338 | Notch-2 | GlcNAcβ-3Fucα-Thr | - | - |
| 339 | Notch-3 | Galβ-4GlcNAcβ-3Fucα-Thr-DH | - | - |
| 340 | GSC-488 | NeuAcα-3Galβ-3GalNAcβ-C30 | - | - |
| 341 | GSC-491 | NeuAcα-3Galβ-3(6-deoxy-6-carboxymethyl)GalNAcβ-C30 | - | - |
| 342 | GSC-489 | SU-6  │ NeuAcα-3Galβ-3GalNAcβ-C30 | - | - |
| 343 | DST | NeuAcα-3Galβ-3GalNAc-DH  │  NeuAcα-6 | - | - |
| 344 | DST-AO | NeuAcα-3Galβ-3GalNAc-AO  │  NeuAcα-6 | - | - |
| 345 | GSC-490 | NeuAcα-3Galβ-3GalNAcβ-C30  │  NeuAcα-6 | - | - |
| 346 | GlcNAcβ-3Fuc-AO | GlcNAcβ-3Fuc-AO | - | - |
| 347 | GlcNAcβ1-2Fuc-AO | GlcNAcβ-2Fuc-AO | - | - |
| 348 | GlcNAcβ1-4Fuc-AO | GlcNAcβ-4Fuc-AO | - | - |
| 349 | GlcNAcβ-2Man-AO | GlcNAcβ-2Man-AO | - | - |
| ***Polysialyl*** | | | | |
| 350 | SA2(α8) | NeuAcα-8NeuAc-DH | 1265 | 63 |
| 351 | SA3(α8) | NeuAcα-8NeuAcα-8NeuAc-DH | - | - |
| 352 | SA4(α8) | NeuAcα-8NeuAcα-8NeuAcα-8NeuAc-DH | - | - |
| 353 | SA5(α8)* | NeuAcα-8NeuAcα-8NeuAcα-8NeuAcα-8NeuAc-DH | - | - |
| 354 | SA6(α8)* | NeuAcα-8NeuAcα-8NeuAcα-8NeuAcα-8NeuAcα-8NeuAc-DH | - | - |
| 355 | SA7(α8)* | NeuAcα-8NeuAcα-8NeuAcα-8NeuAcα-8NeuAcα-8NeuAcα-8NeuAc-DH | - | - |
| 356 | SA8(α8)* | NeuAcα-8NeuAcα-8NeuAcα-8NeuAcα-8NeuAcα-8NeuAc-8NeuAcα-8NeuAc-DH | - | - |
| 357 | SA9(α8)* | NeuAcα-8NeuAcα-8NeuAcα-8NeuAcα-8NeuAcα-8NeuAc-8NeuAcα-8NeuAc-8NeuAcα-DH | - | - |
| 358 | SA10(α8)* | NeuAcα-8NeuAcα-8NeuAcα-8NeuAcα-8NeuAcα-8NeuAcα-8NeuAcα-8NeuAcα-8NeuAcα-8NeuAc-DH | - | - |
| ***Glycosaminoglycan*** | | | | |
| 359 | HA-S4* | GlcAβ-3GlcNAcβ-4GlcAβ-3GlcNAc-DH | - | - |
| 360 | HA-S14* | GlcAβ-3GlcNAcβ-4GlcAβ-3GlcNAcβ-4GlcAβ-3GlcNAcβ-4GlcAβ-3GlcNAcβ-4GlcAβ-3GlcNAcβ-4GlcAβ-3GlcNAcβ-4GlcAβ-3GlcNAc-DH | - | - |
| 361 | Hep-Di IS | ΔUA-4GlcNS-DH  │ │ SU-2 │  SU-6 | - | - |
| 362 | Hep-Di-IS-AO | ΔUA-4GlcNS-AO  │ │ SU-2 │  SU-6 | - | - |
| 363 | CSA-4* | ΔUA-3GalNAcβ-4GlcAβ-3GalNAc-DH  │ │  SU-4 SU-4 | - | - |
| 364 | CSA-14* | ΔUA-3GalNAcβ-4GlcAβ-3GalNAcβ-4GlcAβ-3GalNAcβ-4GlcAβ-3GalNAcβ-4GlcAβ-   │ │ │ │  SU-4 SU-4 SU-4 SU-4  -3GalNAcβ-4GlcAβ-3GalNAcβ-4GlcAβ-3GalNAc-DH  │ │ │  SU-4 SU-4 SU-4 | - | - |
| 365 | CSB-4* | ΔUA-3GalNAcβ-4IdoAα-3GalNAc-DH  │ │  SU-4 SU-4 | - | - |
| 366 | CSB-14* | ΔUA-3GalNAcβ-4IdoAα-3GalNAcβ-4IdoAα-3GalNAcβ-4IdoAα-3GalNAcβ-4IdoAα-   │ │ │ │  SU-4 SU-4 SU-4 SU-4    -3GalNAcβ-4IdoAα-3GalNAcβ-4IdoAα-3GalNAc-DH  │ │ │  SU-4 SU-4 SU-4 | - | - |
| 367 | CSC-4* | ΔUA-3GalNAcβ-4GlcAβ-3GalNAc-DH  │ │  SU-6 SU-6 | - | - |
| 368 | CSC-14* | ΔUA-3GalNAcβ-4GlcAβ-3GalNAcβ-4GlcAβ-3GalNAcβ-4GlcAβ-3GalNAcβ-4GlcAβ-   │ │ │ │   SU-6 SU-6 SU-6 SU-6  -3GalNAcβ-4GlcAβ-3GalNAcβ-4GlcAβ-3GalNAc-DH  │ │ │  SU-6 SU-6 SU-6 | - | - |
| 369 | Hep-4-AO* | SU-2  │  ΔUA-4GlcNSα-4IdoAα-4GlcNS-AO  │ │ │  SU-6 SU-2 SU-6 | - | - |
| 370 | Hep-14-AO* | SU-2  │  ΔUA-4GlcNSα-4IdoAα-4GlcNSα-4IdoAα-4GlcNSα-4IdoAα-4GlcNSα-4IdoAα   │ │ │ │ │ │ │ │   SU-6 SU-2 SU-6 SU-2 SU-6 SU-2 SU-6 SU-2  -4GlcNSα-4IdoAα-4GlcNSα-4IdoAα-4GlcNS-AO  │ │ │ │ │  SU-6 SU-2 SU-6 SU-2 SU-6 | - | - |
| 371 | HS-S4-AO* | GlcAβ-4GlcNAcα-4GlcAβ-4aMan-AO | - | - |
| ***Homo-oligomers*** | | | | |
| 372 | GN2-AO | GlcNAcβ-4GlcNAc-AO | - | - |
| 373 | GN3 | GlcNAcβ-4GlcNAcβ-4GlcNAc-DH | - | - |
| 374 | GN3-AO | GlcNAcβ-4GlcNAcβ-4GlcNAc-AO | - | - |
| 375 | GN4-AO* | GlcNAcβ-4GlcNAcβ-4GlcNAcβ-4GlcNAc-AO | - | - |
| 376 | GN5-AO* | GlcNAcβ-4GlcNAcβ-4GlcNAcβ-4GlcNAcβ-4GlcNAc-AO | - | - |
| 377 | GN6-AO* | GlcNAcβ-4GlcNAcβ-4GlcNAcβ-4GlcNAcβ-4GlcNAcβ-4GlcNAc-AO | - | - |
| 378 | GN7-AO* | GlcNAcβ-4GlcNAcβ-4GlcNAcβ-4GlcNAcβ-4GlcNAcβ-4GlcNAcβ-4GlcNAc-AO | - | - |
| 379 | GN8-AO* | GlcNAcβ-4GlcNAcβ-4GlcNAcβ-4GlcNAcβ-4GlcNAcβ-4GlcNAcβ-4GlcNAcβ-4GlcNAc-AO | - | - |
| 380 | Man4(β4) | Manβ-4Manβ-4Manβ-4Man-DH | - | - |
| 381 | Man6(β4) | Manβ-4Manβ-4Manβ-4Manβ-4Manβ-4Man-DH | - | - |
| 382 | Xyl5(β4) | Xylβ-4Xylβ-4Xylβ-4Xylβ-4Xyl-DH | - | - |
| 383 | Xyl6(β4) | Xylβ-4Xylβ-4Xylβ-4Xylβ-4Xylβ-4Xyl-DH | - | - |
| 384 | Ara6(α5) | Araα-5Araα-5Araα-5Araα-5Araα-5Ara-DH | - | - |
| 385 | Ara7(α5) | Araα-5Araα-5Araα-5Araα-5Araα-5Araα-5Ara-DH | - | - |
| 386 | Nigerose-AO | Glcα-3Glc-AO | - | - |
| 387 | Malto-2-AO | Glcα-4Glc-AO | - | - |
| 388 | Malto-4-AO | Glcα-4Glcα-4Glcα-4Glc-AO | - | - |
| 389 | Malto-6-AO | Glcα-4Glcα-4Glcα-4Glcα-4Glcα-4Glc-AO | - | - |
| 390 | Malto-7-AO | Glcα-4Glcα-4Glcα-4Glcα-4Glcα-4Glcα-4Glc-AO | - | - |
| 391 | Malto-8-AO* | Glcα-4Glcα-4Glcα-4Glcα-4Glcα-4Glcα-4Glcα-4Glc-AO | - | - |
| 392 | Malto-9-AO* | Glcα-4Glcα-4Glcα-4Glcα-4Glcα-4Glcα-4Glcα-4Glcα-4Glc-AO | - | - |
| 393 | Malto-10-AO* | Glcα-4Glcα-4Glcα-4Glcα-4Glcα-4Glcα-4Glcα-4Glcα-4Glcα-4Glc-AO | - | - |
| 394 | Malto-11-AO* | Glcα-4Glcα-4Glcα-4Glcα-4Glcα-4Glcα-4Glcα-4Glcα-4Glcα-4Glcα-4Glc-AO | - | - |
| 395 | Malto-12-AO* | Glcα-4Glcα-4Glcα-4Glcα-4Glcα-4Glcα-4Glcα-4Glcα-4Glcα-4Glcα-4Glcα-4Glc-AO | - | - |
| 396 | Malto-13-AO* | Glcα-4Glcα-4Glcα-4Glcα-4Glcα-4Glcα-4Glcα-4Glcα-4Glcα-4Glcα-4Glcα-4Glcα-4Glc-AO | - | - |
| 397 | Dext-2-AO | Glcα-6Glc-AO | - | - |
| 398 | Dext-3-AO | Glcα-6Glcα-6Glc-AO | - | - |
| 399 | Dext-4-AO | Glcα-6Glcα-6Glcα-6Glc-AO | - | - |
| 400 | Dext-5-AO* | Glcα-6Glcα-6Glcα-6Glcα-6Glc-AO | - | - |
| 401 | Dext-6-AO* | Glcα-6Glcα-6Glcα-6Glcα-6Glcα-6Glc-AO | - | - |
| 402 | Dext-8-AO* | Glcα-6Glcα-6Glcα-6Glcα-6Glcα-6Glcα-6Glcα-6Glc-AO | - | - |
| 403 | Dext-9-AO* | Glcα-6Glcα-6Glcα-6Glcα-6Glcα-6Glcα-6Glcα-6Glcα-6Glc-AO | - | - |
| 404 | Dext-10-AO* | Glcα-6Glcα-6Glcα-6Glcα-6Glcα-6Glcα-6Glcα-6Glcα-6Glcα-6Glc-AO | - | - |
| 405 | Dext-11-AO* | Glcα-6Glcα-6Glcα-6Glcα-6Glcα-6Glcα-6Glcα-6Glcα-6Glcα-6Glcα-6Glc-AO | - | - |
| 406 | Dext-12-AO* | Glcα-6Glcα-6Glcα-6Glcα-6Glcα-6Glcα-6Glcα-6Glcα-6Glcα-6Glcα-6Glcα-6Glc-AO | - | - |
| 407 | Dext-13-AO* | Glcα-6Glcα-6Glcα-6Glcα-6Glcα-6Glcα-6Glcα-6Glcα-6Glcα-6Glcα-6Glcα-6Glcα-6Glc-AO | - | - |
| 408 | Lam-2-AO | Glcβ-3Glc-AO | - | - |
| 409 | Lam-3-AO | Glcβ-3Glcβ-3Glc-AO | - | - |
| 410 | Lam-4-AO | Glcβ-3Glcβ-3Glcβ-3Glc-AO | - | - |
| 411 | Lam-5-AO | Glcβ-3Glcβ-3Glcβ-3Glcβ-3Glc-AO | - | - |
| 412 | Lam-6-AO* | Glcβ-3Glcβ-3Glcβ-3Glcβ-3Glcβ-3Glc-AO | - | - |
| 413 | Lam-7-AO | Glcβ-3Glcβ-3Glcβ-3Glcβ-3Glcβ-3Glcβ-3Glc-AO | - | - |
| 414 | Curd-8-AO* | Glcβ-3Glcβ-3Glcβ-3Glcβ-3Glcβ-3Glcβ-3Glcβ-3Glc-AO | - | - |
| 415 | Curd-9-AO* | Glcβ-3Glcβ-3Glcβ-3Glcβ-3Glcβ-3Glcβ-3Glcβ-3Glcβ-3Glc-AO | - | - |
| 416 | Curd-10-AO* | Glcβ-3Glcβ-3Glcβ-3Glcβ-3Glcβ-3Glcβ-3Glcβ-3Glcβ-3Glcβ-3Glc-AO | - | - |
| 417 | Curd-11-AO* | Glcβ-3Glcβ-3Glcβ-3Glcβ-3Glcβ-3Glcβ-3Glcβ-3Glcβ-3Glcβ-3Glcβ-3Glc-AO | - | - |
| 418 | Curd-12-AO* | Glcβ-3Glcβ-3Glcβ-3Glcβ-3Glcβ-3Glcβ-3Glcβ-3Glcβ-3Glcβ-3Glcβ-3Glcβ-3Glc-AO | - | - |
| 419 | Curd-13-AO* | Glcβ-3Glcβ-3Glcβ-3Glcβ-3Glcβ-3Glcβ-3Glcβ-3Glcβ-3Glcβ-3Glcβ-3Glcβ-3Glcβ-3Glc-AO | - | - |
| 420 | Cellobiose-AO | Glcβ-4Glc-AO | - | - |
| 421 | Cello-3-AO | Glcβ-4Glcβ-4Glc-AO | - | - |
| 422 | Cello-4-AO | Glcβ-4Glcβ-4Glcβ-4Glc-AO | - | - |
| 423 | Cello-5-AO* | Glcβ-4Glcβ-4Glcβ-4Glcβ-4Glc-AO | - | - |
| 424 | Cello-6-AO* | Glcβ-4Glcβ-4Glcβ-4Glcβ-4Glcβ-4Glc-AO | - | - |
| 425 | Cello-7-AO* | Glcβ-4Glcβ-4Glcβ-4Glcβ-4Glcβ-4Glcβ-4Glc-AO | - | - |
| 426 | Cello-8-AO* | Glcβ-4Glcβ-4Glcβ-4Glcβ-4Glcβ-4Glcβ-4Glcβ-4Glc-AO | - | - |
| 427 | Cello-9-AO* | Glcβ-4Glcβ-4Glcβ-4Glcβ-4Glcβ-4Glcβ-4Glcβ-4Glcβ-4Glc-AO | - | - |
| 428 | Cello-10-AO* | Glcβ-4Glcβ-4Glcβ-4Glcβ-4Glcβ-4Glcβ-4Glcβ-4Glcβ-4Glcβ-4Glc-AO | - | - |
| 429 | Cello-11-AO* | Glcβ-4Glcβ-4Glcβ-4Glcβ-4Glcβ-4Glcβ-4Glcβ-4Glcβ-4Glcβ-4Glcβ-4Glc-AO | - | - |
| 430 | Cello-12-AO* | Glcβ-4Glcβ-4Glcβ-4Glcβ-4Glcβ-4Glcβ-4Glcβ-4Glcβ-4Glcβ-4Glcβ-4Glcβ-4Glc-AO | - | - |
| 431 | Cello-13-AO* | Glcβ-4Glcβ-4Glcβ-4Glcβ-4Glcβ-4Glcβ-4Glcβ-4Glcβ-4Glcβ-4Glcβ-4Glcβ-4Glcβ-4Glc-AO | - | - |
| 432 | Gentiobiose-AO | Glcβ-6Glc-AO | - | - |
| 433 | Pust-3-AO | Glcβ-6Glcβ-6Glc-AO | - | - |
| 434 | Pust-4-AO | Glcβ-6Glcβ-6Glcβ-6Glc-AO | - | - |
| 435 | Pust-5-AO | Glcβ-6Glcβ-6Glcβ-6Glcβ-6Glc-AO | - | - |
| 436 | Pust-6-AO | Glcβ-6Glcβ-6Glcβ-6Glcβ-6Glcβ-6Glc-AO | - | - |
| 437 | Pust-7-AO* | Glcβ-6Glcβ-6Glcβ-6Glcβ-6Glcβ-6Glcβ-6Glc-AO | - | - |
| 438 | Pust-8-AO* | Glcβ-6Glcβ-6Glcβ-6Glcβ-6Glcβ-6Glcβ-6Glcβ-6Glc-AO | - | - |
| ***Miscellaneous*** | | | | |
| 439 | Gal | Gal-DH | - | - |
| 440 | Gal-AO | Gal-AO | - | - |
| 441 | GalNAc | GalNAc-DH | - | - |
| 442 | GalNAc-AO | GalNAc-AO | - | - |
| 443 | Glc | Glc-DH | - | - |
| 444 | Glc-AO | Glc-AO | - | - |
| 445 | GN | GlcNAc-DH | - | - |
| 446 | GN-AO | GlcNAc-AO | - | - |
| 447 | Man-AO | Man-AO | - | - |
| 448 | Fuc | Fuc-DH | - | - |
| 449 | Fuc-AO | Fuc-AO | - | - |
| 450 | NeuAc | NeuAc-DH | - | - |
| 451 | NeuAc-AO | NeuAc-AO | - | - |
| 452 | NeuGc | NeuGc-DH | - | - |
| 453 | NeuGc-AO | NeuGc-AO | - | - |
| 454 | Rha | Rha-DH | - | - |
| 455 | Rha-AO | Rha-AO | - | - |
| 456 | Galα-6Glc-AO | Galα-6Glc-AO | - | - |
| 457 | (6P)-Glc-AO | P-6Glc-AO | - | - |
| 458 | (6P)-Man | P-6Man-DH | - | - |
| 459 | (6P)-Man-AO | P-6Man-AO | - | - |
| 460 | (6P)-Fructose-AO | P-6Fru-AO | - | - |
| 461 | SU-Tyr | SU-Tyr-DH | - | - |
| 462 | SU-Cholesterol | SU-Cholesterol | - | - |
| 463 | GN-Asn | GlcNAcβ-Asn-DH | - | - |
| 464 | Xyl3Glc4 | Xylα-6  │  Glcβ-4Glcβ-4Glcβ-4Glc-DH  │ │ Xylα-6 Xylα-6 | - | - |
| 465 | GSC-284 | GalNAcβ-6Galβ-4Glcβ-Cer36  │  NeuAcα-3 | - | - |
| 466 | GSC-575 | GalNAcβ-4Galβ-3Galβ-C30  │  NeuAcα-3 | - | - |
| 467 | GSC-70 | NeuAcα-6Galβ-6GalNAcβ-4Galβ-4Glcβ-Cer36 | 16451 | 703 |
| 468 | GSC-154 | NeuAcα-3Galβ-4GlcNAcβ-6Galβ-4Glcβ-Cer36  │  Fucα-3 | - | - |
| 469 | GSC-446 | NeuAcα-3Galβ-4GlcNAcβ-6GalNAcα-3Galβ-4Glc-C30 | - | - |
| 470 | GSC-441 | NeuAcα-3Galβ-4GlcNAcβ-6GalNAcα-3Galβ-4Glcβ-C30 | - | - |
| 471 | GSC-384 | NeuAcα-3Galβ-4GlcNAcβ-4GalNAcβ-3Galβ-4Glcβ-C30  │  Fucα-3 | - | - |
| 472 | Glc4(α6,α4,α4) | Glcα-6Glcα-4Glcα-4Glc-DH | - | - |
| 473 | Glc(α6,α4,α4)-AO | Glcα-6Glcα-4Glcα-4Glc-AO | - | - |
| 474 | O1-AO | GlcNAcβ-6  │  Gal-AO  │ GlcNAcβ-3 | - | - |
| 475 | Rutinose-AO | Rhaα-6Glc-AO | - | - |

Asterisks that follow the names of certain probes indicate that predominant components are shown. —, signal less than 500.

^a^Pos, Probe position in the binding chart.

^b^The glycan probes are all lipid-linked neoglycolipids (NGLs) or glycosylceramides and are from the collection assembled in the course of research in the

Glycosciences Laboratory. DH, NGLs prepared from reducing oligosaccharides by reductive amination with the amino lipid, 1,2-dihexadecyl-*sn*-glycero-3-phosphoethanolamine (DHPE), DH-NGLs; AO, NGLs prepared from reducing oligosaccharides by oxime ligation with an aminooxy (AO) functionalized DHPE (Liu et al., Chem. Biol. 14, 847–859, 2007); Cer, natural glycolipids with various ceramide moieties; Cer36 and Cer42, synthetic glycolipids with ceramide having a total of 32 and 42 carbon atoms, respectively; C30, a synthetic lipid [2-(tetradecyl)hexadecanol] with 30 carbon atoms.

^c^Numerical scores for the binding signals are shown as means of duplicate spots at 5 fmol per spot.

^d^Difference of signal intensities of duplicated spots of each glycan probe.

**Supplementary Table S2 -** Supplementary glycan microarray document based on MIRAGE guidelines (doi: [10.3762/mirage.3](http://www.beilstein-institut.de/en/projects/mirage/guidelines#glycan_microarrays)).

| **Classification** | **Guidelines** |
| --- | --- |
| 1. **Sample: Glycan Binding Sample** | |
| Description of Sample | Sample name: monoclonal antibody L2A5 (IgM)  Origin: mouse hybridoma  Method of preparation:  The preparation of L2A5 monoclonal antibody is described in the *Materials and Methods* section of the main text. |
| Sample modifications | Not relevant. |
| Assay protocol | Please see *Materials and* *Methods* section in the main text. |
| **2.** **Glycan Library** | |
| Glycan description for defined glycans | Two glycan microarrays were used:  1) Newly developed microarray (in house designation “DA-NGL microarray”) consisting of sialylated and mucin *O*-glycan core probes shown in **Fig. 10 and** **Table 3**.  2) A microarray of sequence-defined lipid-linked glycan probes. Names and structures are in **Supplementary Table S2**. They consist of 475 probes described earlier ([Palma et al., BBRC. 2011](https://www.ncbi.nlm.nih.gov/pubmed/21527252)). These are a sub-set of a recently generated large screening microarray containing around 900 glycan probes (in-house designation “Array Sets 42-56”, which will be published elsewhere). |
| Glycan description for undefined glycans | Not relevant. |
| Glycan modifications | DA, NGLs prepared using a new aldehyde-terminating lipid reagent to conjugate with amino-terminating glycans (detailed description will be published elsewhere); Gly, glycine; DH, NGLs, prepared from reducing oligosaccharides by reductive amination with the amino lipid, 1,2-dihexadecyl-*sn*-glycero-3-phosphoethanolamine [(DHPE) [(Chai et al., Methods Enzymol. 2003)](https://www.ncbi.nlm.nih.gov/pubmed/12968363)]; AO, NGLs prepared from reducing oligosaccharides by oxime ligation with an aminooxy-functionalized DHPE [(AOPE) [(Liu et al., Chem. Biol. 2007)](https://www.ncbi.nlm.nih.gov/pubmed/17656321)].  For full description on the definition of lipid moieties of the glycan probes please see <https://glycosciences.med.ic.ac.uk/docs/lipids.pdf> |
| 1. **3.** **Printing Surface; e.g., Microarray Slide** | |
| Description of surface | Nitrocellulose-coated glass microarray slides. |
| Manufacturer | 16-pad UniSart® 3D Microarray Slide from Sartorius (Goettingen, Germany) |
| Custom preparation of surface | Not relevant. |
| Non-covalent Immobilisation | The lipid-linked oligosaccharide probes were formulated as liposomes by adding carrier lipids, 1,2-dihexanoyl-*sn*-glycero-3-phosphocholine (DHPC) and cholesterol ([Liu et al., Methods Mol. Biol. 2012](https://www.ncbi.nlm.nih.gov/pubmed/22057521)) for arraying and non-covalent immobilisation on nitrocellulose-coated glass slides. |
| **4. Arrayer (Printer)** | |
| Description of Arrayer | Nano-Plotter 2.1 (GeSiM, Radeberg, Germany). |
| Dispensing mechanism | Non-contact liquid delivery with four dispensing tips. |
| Glycan deposition | Approximately 0.33 nl was printed per spot.  Each glycan probes was printed at 2 levels (2 and 5 fmol per spot), in duplicate. |
| Printing conditions | The printing solutions were aqueous-based. Printing was performed at room temperature and relative humidity of 58%.  The NGL printing solutions contained 100 pmol/μl of DHPC and cholesterol (both from SIGMA) as lipid carriers in addition to the lipid-linked glycan probes. The concentrations of the NGL probes were 5 and 15 pmol/μl for the 2 and 5 fmol per spot levels, respectively.  The printing solutions also contained Cy3 NHS ester (GE Healthcare) at 20 ng/ml (26 fmol/μl) as a marker to monitor the printing process. |
| 1. **5.** **Glycan Microarray with “Map”** | |
| Array layout | The arrayed slides contained 16 identical pads (subarrays). Each pad was set up for printing 64 probes maximum, each at 2 levels in duplicate (four spots for one probe in a row); 256 spots (16x16) in total for 64 probes. |
| Glycan identification and quality control | Quality control of the DA-NGL microarray was carried out with: biotinylated plant lectins - Wheat Germ Agglutinin (WGA), *Vicia Villosa* Lectin (VVL) and *Sambucus nigra* lectin (SNL) (Vector Laboratories); Fc-tagged-MGL(CLEC10A) (R&D systems); and monoclonal anti-STn antibody (clone 3F1) (SBH Biosciences).  Quality control of the DA-NGL microarray was carried out with: biotinylated plant lectins - *Ricinus Communis* Agglutinin I (RCA120), *Aleuria aurantia* lectin (AAL), Concanavalin A (ConA) and WGA, (Vector Laboratories); sialic-binding simian virus 40 VP1 ([Campanero-Rhodes et al., J Virol. 2007](https://www.ncbi.nlm.nih.gov/pubmed/17855525)); and short fiber knob protein of human adenovirus 52 ([Lenman et al., Proc Natl Acad Sci U S A. 2018](https://www.ncbi.nlm.nih.gov/pubmed/29674446)).  These data will be described elsewhere. |
| 1. **6. Detector and Data Processing** | |
| Scanning hardware | GenePix 4300A (Molecular Devices, ) |
| Scanner settings | Scanning resolution: 10 μm / pixel (this resolution is adequate for the sizes of sample spots)  Laser channel: Red (scan wavelength 635 nm)  PMT: 350  Scan powers: 10%, 20% or 90% to achieve maximum signal without spot saturation. |
| Image analysis software | GenePix® Pro 7 (Molecular Devices) |
| Data processing | The .gpr file was entered into an in-house microarray database using software (designed by Mark Stoll, <http://www.beilstein-institut.de/en/publications/proceedings/glyco-2009>) for data processing. No particular normalisation method or statistical analysis was used. |
| **7.** **Glycan Microarray Data Presentation** | |
| Data presentation | The microarray binding results are in **Fig. 10** and **Table 3** and in **Supplementary Figure S3** and **Table S1**. |
| 1. **8.** **Interpretation and** **Conclusion from Microarray Data** | |
| Data interpretation | No software or algorithms were used to interpret processed data. |
| Conclusions | L2A5 binds strongly to the short mucin core STn antigen disaccharide probes (NeuAcα-6GalNAcα-Ser/Thr-DA) and weakly to α2-6-linked sialyl core-1 probes (Galβ-3(NeuAcα-6)GalNAcα-Ser-/Thr-DA). L2A5 could also bind to the STn prepared as AO-NGL (NeuAcα-6GalNAcβ-AO). There was weak, but detectable, binding to α-6-sialyllactose probes (NeuAcα-6Galβ-4Glcβ-Gly-DA and NeuAcα-6Galβ-4Glcβ-AO) and to the NeuAcα-8NeuAc-DH probe.  An incidental finding of uncertain significance was binding to a synthetic unnatural sialylated glycolipid with the sequence NeuAcα-6Galβ-6GalNAcβ-4Galβ-4Glcβ-. |
